# Aerobic Denitrification and Heterotrophic Sulfur Oxidation in the Genus *Halomonas* Revealed by Six Novel Species Characterizations and Genome-Based Analysis

**DOI:** 10.3389/fmicb.2021.652766

**Published:** 2021-03-18

**Authors:** Liping Wang, Zongze Shao

**Affiliations:** ^1^School of Environment, Harbin Institute of Technology, Harbin, China; ^2^Key Laboratory of Marine Genetic Resources, Third Institute of Oceanography, Ministry of Natural Resources, State Key Laboratory Breeding Base of Marine Genetic Resources, Fujian Key Laboratory of Marine Genetic Resources, Xiamen, China; ^3^Laboratory for Marine Biology and Biotechnology, Pilot National Laboratory for Marine Science and Technology, Qingdao, China

**Keywords:** *Halomonas*, taxonomy, novel species, aerobic denitrification, heterotrophic sulfur oxidation

## Abstract

Bacteria of *Halomonas* are widely distributed in various environments and play a substantial role in the nutrient cycle. In this report, 14 strains capable of aerobic denitrification and heterotrophic sulfur oxidation were isolated from different habitats. Based on the phenotypic, genotypic, and chemotaxonomic analyses, these strains were considered to represent six novel species of the genus *Halomonas*, for which the names *Halomonas zhangzhouensis* sp. nov. type strain CXT3-11^T^ ( = MCCC 1A11036^T^ = KCTC 72087^T^), *Halomonas aerodenitrificans* sp. nov. CYD-9^T^ ( = MCCC 1A11058^T^ = KCTC 72088^T^), *Halomonas sulfidoxydans* sp. nov. CYN-1-2^T^ ( = MCCC 1A11059^T^ = KCTC 72089^T^), *Halomonas ethanolica* sp. nov. CYT3-1-1^T^ ( = MCCC 1A11081^T^ = KCTC 72090^T^), *Halomonas sulfidivorans* sp. nov. NLG_F1E^T^ ( = MCCC 1A13718^T^ = KCTC 72091^T^), and *Halomonas tianxiuensis* sp. nov. BC-M4-5^T^ ( = MCCC 1A14433^T^ = KCTC 72092^T^) are proposed. Intriguingly, they formed a unique group with 11 other species designated as the “*H. desiderata* group.” To better understand their featured metabolisms, genes involved in denitrification and sulfur oxidation were analyzed, along with 193 other available genomes of the whole genus. Consistently, complete denitrification pathways were confirmed in the “*H. desiderata* group,” in which *napA*, *narG*, *nirS*, *norB*, and *nosZ* genes coexist. Their nitrite reductase NirS formed a unique evolutionary lineage, distinguished from other denitrifiers in *Halomonas*. In addition, diverse occurrence patterns of denitrification genes were also observed in different phylogenetic clades of *Halomonas*. With respect to sulfur oxidation, *fccAB* genes involved in sulfide oxidation commonly exist in the “*H. desiderata* group,” while *sqr* genes are diverse and can be found in more species; *sqr* genes co-occurred with *fccAB* in eight strains of this study, contributing to more active sulfide oxidation. Besides, the *tsdA* gene, which encodes an enzyme that oxidizes thiosulfate to tetrathionate, is ubiquitous in the genus *Halomonas*. The widespread presence of *sqr/fccAB*, *pdo*, and *tsdA* in *Halomonas* suggests that many *Halomonas* spp. can act as heterotrophic sulfur oxidizers. These results provide comprehensive insights into the potential of denitrification and sulfur oxidation in the whole genus of *Halomonas*. With regard to the global distribution of *Halomonas*, this report implies their unneglectable role in the biogeochemical cycle.

## Introduction

The genus *Halomonas*, which was established in 1980, belongs to the Halomonadaceae family of the order Oceanospirillales, within the class Gammaproteobacteria. They are a group of Gram-stain-negative, halophilic or halotolerant, aerobic or facultatively anaerobic, and non-sporulated bacteria (Vreeland et al., 1980). At present, more than 100 species in the genus *Halomonas* have been described^1^. As a cosmopolitan taxon, members of the genus *Halomonas* widely inhabit diverse saline environments, such as salt and soda lakes, saline soil, solar salterns, sea ice, seafood, marine invertebrates, effluents, seawater, sea basins, hadal, and hydrothermal vent environments (Kaye and Baross, 2000; Kaye et al., 2004, 2011; Biddle et al., 2005; Inagaki et al., 2006; Arahal et al., 2007; Kim et al., 2013; Nunoura et al., 2015; Salazar et al., 2016; Sanz-Sáez et al., 2020).

The *Halomonas* bacteria have been described as opportunitrophic microbes, which can readily adapt to various environments and exploit spatially and temporally variable resources, although they are not generally observed to dominate a particular niche (Polz et al., 2006; Kaye et al., 2011; Singer et al., 2011). *Halomonas* is one of the most abundant genera in the ocean and is commonly isolated, and its success may be attributed to its metabolic and physiological versatility. For instance, some *Halomonas* species could produce compatible solutes (ectoine, glycine betaine, and hydroxyectoine), biopolymers [exopolysaccharides (EPSs) and polyhydroxyalkanoate (PHA)], and biosurfactants (Cánovas et al., 2000; Amjres et al., 2011; Schwibbert et al., 2011; Kawata et al., 2014); degrade aromatic compounds (García et al., 2004); and oxidize arsenite (Lin et al., 2012; Hamamura et al., 2014), Fe(II), Mn(II) (Homann et al., 2009; Dong et al., 2014), and sulfide (Boltianskaia et al., 2007; Zhang et al., 2020); and are capable of denitrification (González-Domenech et al., 2010; Guo et al., 2013).

Biological denitrification is a facultative respiratory pathway in which nitrate, nitrite, nitric oxide, and nitrous oxide are stepwise reduced to dinitrogen gas, each catalyzed by at least one specific metalloenzyme, with concomitant energy conservation (Zumft, 1997). Aerobic denitrification was first reported in *Thiosphaera pantotropha* (now known as *Paracoccus denitrificans*), where oxygen and nitrate were simultaneously utilized as electron acceptors (Robertson and Kuenen, 1984). This important process has been intensively studied during the past three decades. The ability of aerobic denitrification is widely distributed among prokaryotic taxa such as *Paracoccus*, *Pseudomonas*, *Marinobacter*, and *Achromobacter*, as well as some eukaryotes in various natural and engineered ecosystems (Ji et al., 2015; He et al., 2016; Liu et al., 2016b, 2018; Luque-Almagro et al., 2017). Most of our current knowledge about aerobic denitrification is related to the nitrogen removal characteristics of single strains, the expression patterns of functional genes, and practical applications, as well as electron transfer (Yang et al., 2020). Denitrification has been recognized as an important functional trait of the genus *Halomonas*, as many species have been described as denitrifiers, and some even as aerobic denitrifiers (González-Domenech et al., 2010). Most studies on denitrifying species within the genus *Halomonas* are merely restricted to their phylogenetic analysis and phenotypic characterization. Knowledge of their denitrification, especially aerobic denitrification mechanism is quite limited, and is based on only a few functional genes obtained through PCR amplification. There is a lack of genomic information with respect to the denitrification pathway in *Halomonas*.

Aerobic denitrification has been widely applied in biological nitrogen removal systems due to their high growth rate and their excellent nitrate removal ability. Recently, heterotrophic sulfide-oxidizing nitrate-reducing bacteria, such as *Pseudomonas* C27 (Chen et al., 2013; Guo et al., 2019; Zhang et al., 2020), have attracted increasing attention. Sulfide oxidation of heterotrophic denitrifiers has also been observed in *Halomonas mongoliensis* and *Halomonas kenyensis*, which can oxidize sulfide in the presence of acetate as the carbon source and nitrate or nitrous oxide as the electron acceptor (Boltianskaia et al., 2007). Several studies have shown that *Halomonas* spp. dominate in bioreactors and play important roles in the removal of sulfide and nitrate (Liu et al., 2016a; Zhang et al., 2020). However, their sulfur oxidation mechanisms are unclear.

In this study, we isolated 14 *Halomonas* strains from various environments, among which six putative novel species were proposed (Table 1). All these 14 strains could perform aerobic denitrification and heterotrophic sulfide oxidation. In order to establish their robust taxonomic status, polyphasic taxonomic analysis was carried out. To gain more insight into their functional profiles, genome-based analysis of these 14 strains and 193 other publicly available *Halomonas* genomes was carried out, based on nitrogen metabolism, especially denitrification and the sulfur oxidation pathway. The objectives of this study are to (i) establish their robust taxonomic status, (ii) analyze their aerobic denitrification and sulfide oxidation performance, and (iii) uncover the nitrogen and sulfur metabolic pathways within the genus *Halomonas*.

**TABLE 1:** The detailed information for all the strains isolated in this study.

MCCC no.	Original no.	NCBI accession	Geographic origin	Isolation source	Depth (m)	Novel species
1A05748	NH62J	JABFTQ000000000	South China Sea	Deep sea sediments	1,000	
1A05775	NH67M	JABFTR000000000	South China Sea	Deep sea sediments	1,000	
1A05776	NH67N	JABFTS000000000	South China Sea	Deep sea sediments	1,000	
1A11036	CXT3-11	JABFTT000000000	China: Zhangzhou City	Sediments from shrimp culture pond	5	Yes
1A11057	CYD-5	JABFTU000000000	China: Taiwan Strait	Coastal water	5	
1A11058	CYD-9	JABFTV000000000	China: Taiwan Strait	Coastal water	5	Yes
1A11059	CYN-1-2	CP053381	China: Taiwan Strait	Surface sediments in coastal sea	0	Yes
1A11062	CYQ2-2-1	JABFTW000000000	China: Xiamen City	Sewage from municipal wastewater treatment plant	2	
1A11081	CYT3-1-1	JABFTX000000000	China: Zhangzhou City	Sediments from shrimp culture pond	5	Yes
1A13718	NLG_F1E	CP053383	Pacific Ocean	Deep sea sediments	1618	Yes
1A14433	BC-M4-5	CP035042	Northwest Indian Ocean	Sulfide from Tianxiu hydrothermal vents	3440	Yes
1A17486	BC-M3-7	JABFTY000000000	Northwest Indian Ocean	Sulfide chimney from Daxi hydrothermal vents	3450	
1A17488	BC-M4-4	JABFTZ000000000	Northwest Indian Ocean	Sulfide from Tianxiu hydrothermal vents	3440	
1A17499	BC-M2-4	JABFUA000000000	Northwest Indian Ocean	Sulfide from Tianxiu hydrothermal vents	3440	

*NCBI, National Center for Biotechnology Information.*

## Results and Discussion

### Phylogeny of 14 Strains Based on Conserved Genes and Genome Sequences

Based on 16S rRNA, *gyrB*, and *rpoD* gene sequences, the phylogenetic positions of the 14 strains of *Halomonas* were analyzed, allocating them into six potential novel species, represented by strains MCCC 1A11036^T^, MCCC 1A11058^T^, MCCC 1A11059^T^, MCCC 1A11081^T^, MCCC 1A14433^T^, and MCCC 1A13718^T^. The former five strains shared the highest 16S rRNA gene sequence similarities with *Halomonas lactosivorans* KCTC 52281^T^ of 98.9, 98.5, 99.4, 98.9, and 99.3%; MCCC 1A13718^T^ shared the highest similarities with *Halomonas saliphila* LCB169^T^ of 98.8%. For the *gyrB* and *rpoD* gene sequences, the six novel species showed 85.3–95.9 and 87.3–97% similarities with the three most closely related species (*H. desiderata* DSM 9502^T^, *Halomonas daqingensis* CGMCC 1.6443^T^, and *H. lactosivorans* KCTC 52281^T^). Therefore, the *gyrB* gene seems to have a much higher discriminatory power, which has been proposed as a marker gene for species delineation within *Halomonas* (de la Haba et al., 2012).

**FIGURE 1:**
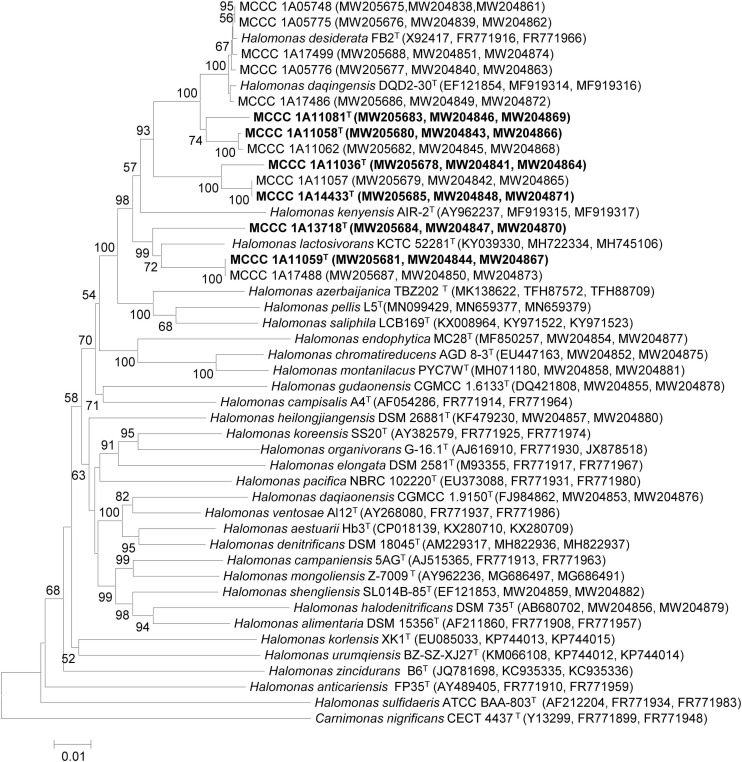
Neighbor-joining tree based on the concatenated 16S rRNA, *gyrB*, and *rpoD* gene sequences showing the phylogenetic positions of six novel strains within the genus *Halomonas*. The type strain *Carnimonas nigrificans* CECT 4437^T^ was used as outgroup. Bootstrap values (>50%) are shown at branch points. Bar, 0.01 nucleotide substitution rate (Knuc) units. The phylogeny was inferred using MEGA version 7 and 1,000 bootstrap replicates. Each type strain is marked by a superscript capital T.

**FIGURE 2:**
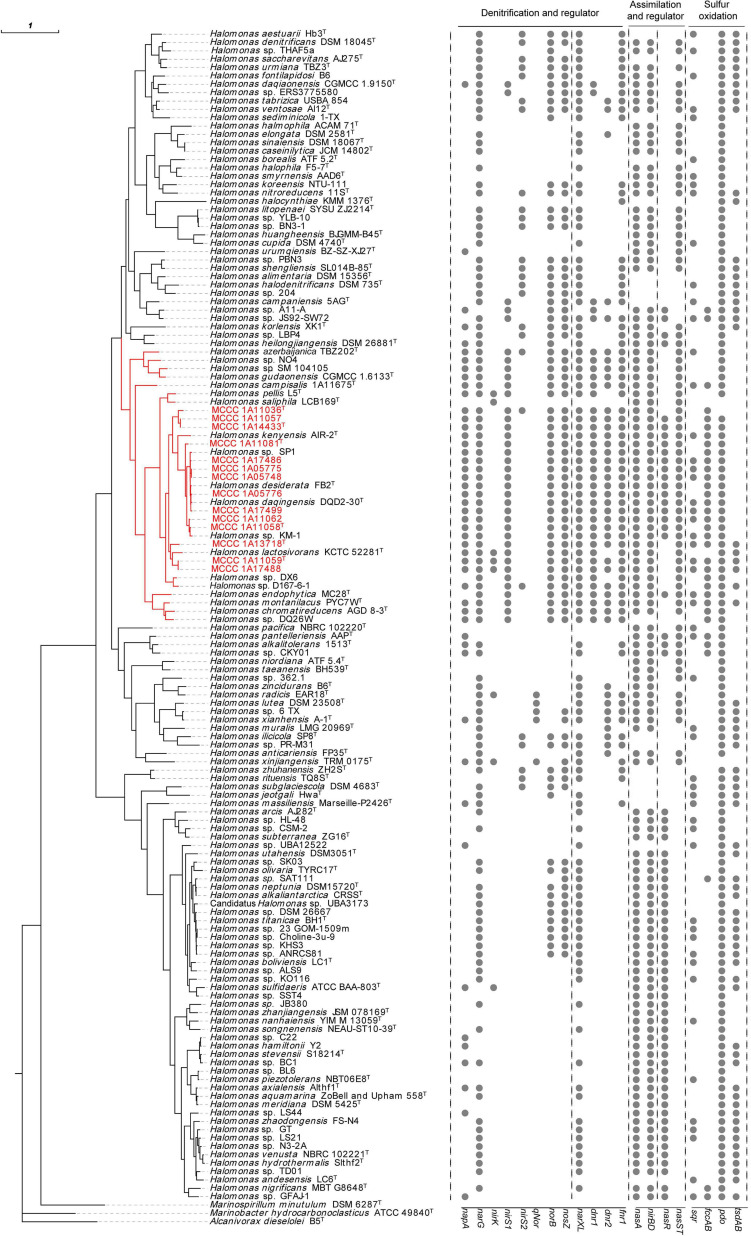
Genome-based phylogenomic tree of the 14 strains along with 125 other representative reference genomes in the genus *Halomonas* and the distribution of genes for nitrogen and sulfur metabolism. The phylogenomic tree was constructed using PhyloPhlAn 3.0. Strains shown in red are organisms isolated in this study. The visualization, annotation, and management of the phylogenomic trees were performed using the web-based tool evolview v3. The type strain *Alcanivorax dieselolei* B5^T^ was used as outgroup. The branch labeled in red indicates the “*H. desiderata* group.” The 16 rightmost columns represent the distribution of key functional genes for the denitrification, regulation, and sulfur oxidation pathways.

These 14 strains formed a closely related group with type strains of *H. desiderata* DSM 9502^T^, *H. daqingensis* CGMCC 1.6443^T^, *H. lactosivorans* KCTC 52281^T^, *Halomonas kenyensis* DSM 17331^T^, *H. saliphila* LCB169^T^, and *Halomonas pellis* L5^T^ in the phylogenetic tree based on the concatenation of 16S rRNA, *gyrB*, and *rpoD* gene sequences, as well as other species, including *Halomonas azerbaijanica* TBZ202^T^, *Halomonas campisalis* A4^T^, *Halomonas gudaonensis* CGMCC 1.6133^T^, *Halomonas endophytica* MC28^T^, *Halomonas montanilacus* PYC7W^T^, and *Halomonas chromatireducens* AGD8-3^T^ (Figure 1). This group could be defined as the “*H. desiderata* group,” which is composed of at least 18 species. Phylogenomic trees were also constructed using both PhyloPhlAn and UBCG software based on genome sequences, and the phylogenetic relationships of “*H. desiderata* group” strains were further verified (Figure 2 and Supplementary Figures 1,2).

### Genome Properties and Genetic Relatedness of the 14 Strains

The general genome properties of the 14 newly sequenced strains are summarized in Supplementary Table 1. Complete genome sequences of strains MCCC 1A11059^T^, MCCC 1A13718^T^, and MCCC 1A14433^T^ and draft genomes for 11 other strains were obtained. Their genome sizes ranged from 4.41 to 5.08 Mb; the number of contigs ranged from 1 to 96; the G+C content ranged from 63.3% (MCCC 1A11036^T^) to 66.2% (MCCC 1A17488), which is within the range of G+C values for *Halomonas* (Kim et al., 2013).

**FIGURE 3:**
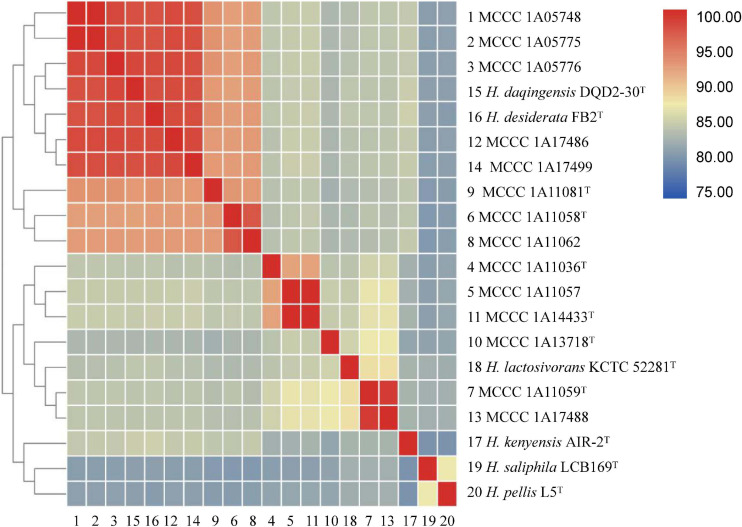
A matrix of the average nucleotide identity between the six novel strains and other closely related species within the genus *Halomonas*. The ANIb values between each pair of genomes were calculated as described in the *Materials and Methods* section. The numerical values underlying this matrix are provided in Supplementary Table 2.

Pairwise digital DNA–DNA hybridization (dDDH) and average nucleotide identity (ANI) values (ANIb, fastANI, and OrthoANI) were calculated to identify the genomic similarities of the 14 strains to six closely related species within the genus *Halomonas* (as shown in Figure 3 and Supplementary Table 2). For strains MCCC 1A11036^T^, MCCC 1A11058^T^, MCCC 1A11059^T^, MCCC 1A11081^T^, MCCC 1A13718^T^, and MCCC 1A14433^T^, the dDDH and ANIb values between them and closely related species were below the 70% (Wayne et al., 1987) and 95–96% (Richter and Rosselló-Móra, 2009) threshold values for species definition, ranging at 24.8–53.6 and 84.4–93.2%, respectively, which could represent six novel species of the genus *Halomonas*.

### Phenotypic Characteristics of Six Novel Species

All six of the strains (MCCC 1A11036^T^, MCCC 1A11058^T^, MCCC 1A11059^T^, MCCC 1A11081^T^, MCCC 1A13718^T^, and MCCC 1A14433^T^) were Gram-stain-negative and catalase- and oxidase-positive. Their cells were long rods, motile by means of peritrichous flagella (Supplementary Figure 3). All strains grew well on marine agar (MA), with yellow, circular, and smooth colonies. They could grow at 4–50°C, even up to 55°C for strain MCCC 1A13718^T^. They could tolerate salinity up to 18% (w/v), even up to 20% for strain MCCC 1A13718^T^. In the API ZYM, API 20NE, and API 20E tests, all the strains were analyzed in parallel with other reference species. They displayed similar physiological characteristics. Differences in phenotypic and physiological characteristics among these strains and other closely related species are shown in Table 2 and in the species descriptions.

**TABLE 2:** Differential characteristics of the six novel species and closely related *Halomonas* species.

Characteristic	1	2	3	4	5	6	7	8	9	10	11	12	13
Cell size (μm)	0.5–0.7×1.0–3.0	0.6–1.1×1.7–2.7	0.7–0.8×1.3–2.0	0.6–0.8×1.6–2.4	0.6–0.7×1.7–2.4	0.6–0.7×1.3–2.1	0.7–0.8×1.0–1.2	0.4–0.6×1.0–2.6	0.5–0.7×1.5–2.5	0.4–0.5×1.5–2.5	0.3–0.5×1.0–1.6	0.8–2.0×0.2–0.5	0.4–0.7×1.2–1.7
Optimum temperature (°C)	37–40	37	37–40	37–40	37–40	37–40	30	37–42	36–40	28–37	30	30	30–35
Temperature range (°C)	4–50	4–50	4–50	4–50	4–55	4–50	10–50	10–45	10–55	10–50	10–52	10–45	5–45
Optimum pH	7–8	7–8	7–8	7–8	7–8	7–8	9	9.7	9.5	7–8	8	8	8
pH range	7–10	7–10	6–10	7–10	6–10	6–10	8–10	7–11	7.5–10.6	6–9	6–10	6–10	7–11
Optimum NaCl concentration (%, w/v)	2–8	2–8	2–6	1–10	2–8	2–8	5–10	9	3–7	4–10	10–15	10	3–8
NaCl concentration range (%, w/v)	0–18	0–18	0–18	0–18	0–20	0–18	1–15	1–18	0–13	1–12	0–17	1–17	0–20
**Hydrolysis of:**													
Tween-20	−	+	−	+	−	−	+	+	−^#^	+	−^#^	+	−
Tween-40	−	+	−	+	−	−	+	−	−	+	−^#^	−	−
Tween-60	−	+	−	+	−	−	−	−	−	+	−	−	−
Tween-80	−	+	−	+	−	−	+	−	+	−	−	−	
DNA	−	−	−	−	+	+	+	+	−	−	+^#^	−	+
Gelatin	−	−	+	−	−	−	w^#^	+^#^	−^#^	−	+	−	+
Starch	−	+	−	−	+	+	−	−	−	−	−	−	−
Casein	−	−	−	−	−	−	−^#^	−	−	+	−	−	−
ONPG	−	−	−	−	−	−	−	−	−	+	−	−	−^#^
Voges–Proskauer test	w	w	w	w	+	+	w	w	+	−	−^#^	+	
D-Glucose fermentation	w	w	w	w	w	w	w	w	−	−	w	+	
**API ZYM**													
Esterase lipase (C8)	w	w	w	+	w	−	w	+	w	+	w	nd	w
Lipase (C14)	w	w	w	−	w	w	−	−	w	+	−	nd	w
Leucine aminopeptidase	+	+	+	+	+	+	−	+	+	+	+	nd	+
Cystine aminopeptidase	w	w	w	w	w	w	−	w	w	+	+	nd	w
Trypsin	−	+	w	−	−	−	w	+	w	+	w	nd	w
α-Glucosidase	−	+	+	+	−	+	+	+	w	+	−	nd	+
API 20NE													
Reduction of nitrate	+	+	+	+	+	+	+	+	+	+	−	+	+
Denitrification	+	+	+	+	+	+	+	+	+	−	−	+	−
Arginine dihydrolase	−	−	w	−	−	−	−^#^	−	−	−	−	nd	+
β-Glucosidase (aesculin hydrolysis)	−	−	−	−	−	−	−	−	−	−	+	−	+
Gelatinase	−	−	+	−	−	−	w^#^	+^#^	−^#^	−	+	−	+
**Assimilation of (API 20NE):**													
D-Glucose	w	w	+	w	+	+	+	w	w	+	+	+	+
L-Arabinose	w	−	+	−	+	+	+	−	−	+	+	+	+
D-Mannose	−	−	−	−	−	−	−	−	−	−	+	−	+
D-Mannitol	+	+	+	+	+	+	+	w	−	+	+	−	+
*N*-Acetyl-glucosamine	−	−	−	−	+	−	−	−	−	+	+	nd	+
D-Maltose	−	+	+	+	+	+	+	+	−	+	+^#^	+	+
Potassium gluconate	+	+	+	+	+	+	+	w	w	+	+	−	+
Capric acid	−	−	−	−	−	−	−	−	−	−	−	nd	w
Adipic acid	−	+	+	w	+	+	+	−	−	+	+	nd	−
Malic acid	+	+	+	+	+	+	+	+	−	+	+	−	+
Trisodium citrate	+	+	+	+	+	+	+	+	−	+	+	+	+
Phenylacetic acid	−	−	−	−	−	+	−	−	−	−	+	nd	−
Sulfide oxidation	w	w	+	w	+	w	w	w	+	−	−	−	−
Thiosulfate oxidation	−	−	+	−	+	−	−	−	−	+	−	−	−
DNA G+C content (%)	63.3	64	66	64.5	64.2	63.9	64.9	64.7	63.7	66.7	64.1	63.5	63.6

*Strains: 1, MCCC 1A11036^*T*^; 2, MCCC 1A11058^*T*^; 3, MCCC 1A11059^*T*^; 4, MCCC 1A11081^*T*^; 5, MCCC 1A13718^*T*^; 6, MCCC 1A14433^*T*^; 7, *Halomonas daqingensis* CGMCC 1.6443^*T*^ (Wu et al., 2008); 8, *Halomonas desiderata* DSM 9502^*T*^ (Berendes et al., 1996); 9, *Halomonas kenyensis* DSM 17331^*T*^ (Boltianskaia et al., 2007); 10, *Halomonas lactosivorans* KCTC 52281^*T*^ (Ming et al., 2020); 11, *Halomonas saliphila* LCB169^*T*^ (Gan et al., 2018); 12, *Halomonas pellis* L5^*T*^ (Li et al., 2020); 13, *Halomonas elongata* DSM 2581^*T*^. The data for strains 1–6 and the data from API 20E, API 20NE, and API ZYM tests for strains 7–11 and 13 were obtained from this study. The data for strain 12 were from Li et al. (2020). All strains were Gram-stain-negative, catalase- and oxidase-positive, and negative for hydrolysis of cellulose and urea, H_2_S production, and indole production. For API 20E, all strains were negative for lysine decarboxylase, ornithine decarboxylase, urease, and tryptophan deaminase. For API ZYM, all strains were positive for alkaline phosphatase, esterase, valine aminopeptidase, acid phosphatase, and naphthol-AS-Bl-phosphoamidase and negative for α-chymotrypsin, α-galactosidase, β-galactosidase, β-glucuronidase, β-glucosidase, *N*-acetyl-β-glucosaminidase, α-mannosidase, and α-fucosidase. +, positive; −, negative; w, weakly positive; nd, not determined; #, the results differed with reference.*

The predominant respiratory quinone of these six species was Q-9, with the minor ubiquinone Q-8 also present, in agreement with previous observations in the genus *Halomonas*. Cellular fatty acid composition was determined for the six species in parallel with reference type strains, and the results are detailed in Table 3. The principal fatty acids for all of the strains (>10%) were C_18:1_ *ω*7*c*, C_16:0_, C_19:0_ cyclo *ω*8*c*, summed feature 3 (C_16:1_ *ω*7*c* and/or C_16:1_ *ω*6*c*), and C_12:0_ 3-OH. Although the fatty acid profiles from these strains were similar, their proportions were different from each other. The polar lipids of the six novel strains include diphosphatidylglycerol (DPG), phosphatidylglycerol (PG), phosphatidylethanolamine (PE), and several unidentified phospholipids (PLs) (Supplementary Figure 4). Distinctive features were the presence of aminolipid (AL) in strains MCCC 1A13718^*T*^ and MCCC 1A11059^T^ and the presence of aminophospholipid (APL) and phosphatidylcholine (PC) in strain MCCC 1A11059^T^. Phenotypic and physiological characteristics as well as chemotaxonomic features that could distinguish these six novel strains from other closely related strains are presented in Table 2 and in the species descriptions.

**TABLE 3:** Cellular fatty acid compositions of six novel strains compared with other closely related species of the genus *Halomonas*.

	1	2	3	4	5	6	7	8	9	10	11	12
C_10:0_	3.3	2.4	2.7	2.5	5.2	2.2	2.4	2.0	3.5	3.2	2.7	2.8
C_12:0_	1.5	1.3	3.7	1.3	5.8	2.5	1.0	1.4	−	4.6	3.7	4.8
C_12:0_ iso	−	−	0.4	−	0.3	0.4	−	−	1.0	0.2	−	−
C_12:0_ 3-OH	7.7	6.6	6.3	5.6	**10.0**	5.6	5.8	5.7	4.0	6.1	7.0	**10.3**
C_14:0_	3.9	3.5	0.4	2.7	0.5	1.3	3.3	2.8	3.1	0.3	0.2	0.4
C_16:0_	**19.0**	**14.4**	**20.1**	**13.9**	**19.4**	**22.4**	**12.7**	**15.4**	**20.6**	**20.9**	**20.9**	**28.4**
Summed feature 3*	4.9	5.5	**11.6**	3.8	**13.4**	5.4	3.8	3.3	**10.9**	**11.6**	**15.0**	6.3
C_17:0_ cyclo	1.4	1.5	1.3	0.9	4.3	5.1	1.2	1.1	2.4	1.9	7.1	5.1
C_18:0_	0.6	0.4	0.8	1.5	0.4	0.5	0.7	0.5	0.9	0.3	0.3	0.5
C_18:1_ *ω*7*c*	**40.1**	**50.8**	**48.0**	**56.8**	**32.0**	**25.8**	**50.3**	**47.4**	**45.0**	**42.9**	**31.0**	**16.1**
C_19:0_ cyclo *ω*8*c*	**15.3**	8.8	2.7	8.7	6.7	**25.6**	**16.0**	**17.6**	7.1	**7.5**	**10.3**	**21.8**

Strains: 1, MCCC 1A11036^*T*^; 2, MCCC 1A11058^*T*^; 3, MCCC 1A11059^*T*^; 4, MCCC 1A11081^*T*^; 5, MCCC 1A13718^*T*^; 6, MCCC 1A14433^*T*^; 7, *Halomonas daqingensis* CGMCC 1.6443^*T*^; 8, *Halomonas desiderata* DSM 9502^*T*^; 9, *Halomonas kenyensis* DSM 17331^*T*^; 10, *Halomonas lactosivorans* KCTC 52281^*T*^; 11, *Halomonas saliphila* LCB169^*T*^; 12, *Halomonas elongata* DSM 2581^*T*^. * Summed features represent groups of two or three fatty acids that could not be separated using the MIDI system. Summed feature 3 contained C_16:1_*ω*7*c*/C_16:1_*ω*6*c*. All of the data were obtained from this study; values shown are percentages of total fatty acids. −, percentage was less than 0.5% or fatty acid was not detected. Fatty acids that represented >10.0% are indicated in bold font.

### Performance of Aerobic Denitrification

**FIGURE 4:**
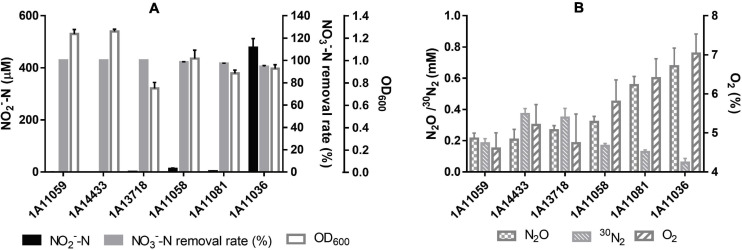
Cell growth and performance of aerobic denitrification by six novel species within 24 h in this study. **(A)** NO_3_^–^ –N removal rate, NO_2_^–^ –N accumulation, and cell OD_600_ with an initial Na^15^NO_3_ concentration of 15 mM. **(B)** ^30^N_2_ and N_2_O production within 24 h as well as final O_2_ percentage, with an initial O_2_ content of 25%. The data are shown as mean value ± SD from three independent measurements.

All six of the novel species can perform denitrification using nitrate or nitrite as the sole nitrogen source, under both aerobic and anaerobic conditions. Their aerobic denitrification performance is shown in Figure 4, where nitrate was used as the sole nitrogen source and the O_2_ percentage in the headspace of the bottles was set at about 25%. As depicted in Figure 4B, their gaseous products were identified to be N_2_ and N_2_O. They exhibited efficient removal ability for nitrate; they could remove 15 mM of nitrate completely within 24 h under aerobic conditions, with the removal rate of NO_3_^–^–N reaching 8.75 mg L^–1^ h^–1^, which is much higher than that of other aerobic denitrifiers (Liu et al., 2018). Besides, the percentage of O_2_ decreased to about 5% within 24 h, suggesting that they could perform co-respiration of nitrate and oxygen during denitrification. Although nitrite accumulated during nitrate reduction, it was removed quickly and was nearly undetectable within 24 h.

Acetate, sodium citrate, glucose, sucrose, lactate, glycerol, mannitol, maltose, succinate, and propionate can be used as carbon sources and electron donors to support cell growth coupling aerobic denitrification for all these strains. Moreover, sodium citrate and succinate were the optimal electron donors observed in this study, with the highest nitrate removal efficiency and the lowest nitrite accumulation, as shown in Supplementary Table 3. Besides, strains MCCC 1A11058^T^, MCCC 1A11081^T^, MCCC 1A13718^T^, and MCCC 1A14433^T^ can also utilize ethanol as electron donor to derive energy for aerobic denitrification.

### Heterotrophic Sulfur Oxidation by the Novel Species

**FIGURE 5:**
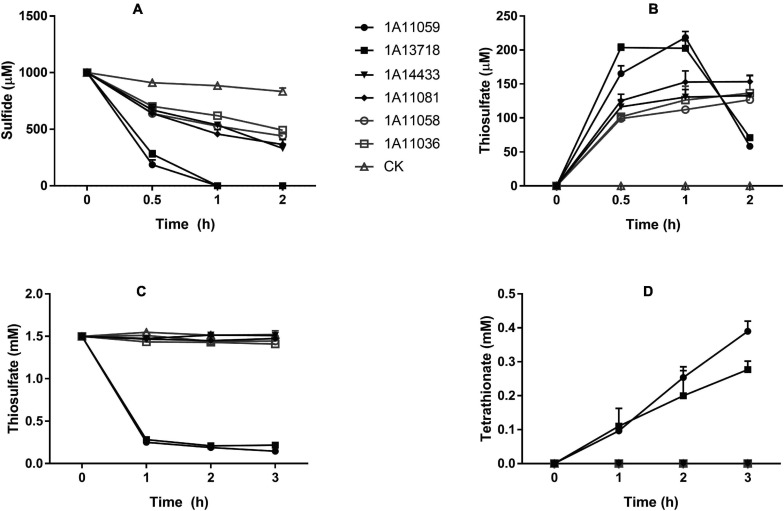
Sulfide and thiosulfate oxidation by six novel strains. **(A)** Sulfide oxidation by six strains with an initial Na_2_S concentration of 1 mM. **(B)** The production of thiosulfate during sulfide oxidation. **(C)** Thiosulfate oxidation by six strains with an initial Na_2_S_2_O_3_ concentration of 1.5 mM. **(D)** The production of tetrathionate during thiosulfate oxidation. CK, control containing no cells. The data are shown as mean value ± SD from three independent measurements.

Another important trait of all these isolates was their ability for heterotrophic sulfide oxidation. As shown in Figure 5A and Supplementary Table 4, strains MCCC 1A11059^T^ and MCCC 1A13718^T^ exhibited the strongest sulfide oxidation ability under aerobic conditions; they could completely oxidize 1 mM of Na_2_S within 1 h, with sulfide oxidation rates reaching 50 and 54 μmol min^–1^ g^–1^ of cell dry weight, respectively, which were comparable with those of other reported heterotrophic sulfide oxidizers (Hou et al., 2018). Thiosulfate was identified as one of the main products; however, no sulfite and sulfate could be detected (Figure 5B). These strains varied in sulfide tolerance; for example, strains MCCC 1A11059^T^ and MCCC 1A13718^T^ could grow and aerobically oxidize at least 7 and 3 mM of Na_2_S, respectively, in the presence of nitrate and sodium citrate; strains MCCC 1A11058^T^ and MCCC 1A11081^T^ could tolerate even 12 mM of Na_2_S, although their sulfide oxidation ability was weak. In the thiosulfate oxidation test, only strains MCCC 1A11059^T^ and MCCC 1A13718^T^ could oxidize thiosulfate rapidly, producing tetrathionate (Figures 5C,D).

Under anaerobic conditions, all six of the novel strains could oxidize sulfide when nitrate was used as the sole electron acceptor; meanwhile, strains MCCC 1A13718^T^ and MCCC 1A11036^T^ could oxidize sulfide using either nitrate or N_2_O as the sole electron acceptor. Several heterotrophic denitrifiers were reported previously to oxidize sulfide or thiosulfate, including *H. kenyensis* AIR-2 and *Halomonas mongoliensis* Z-7009. The former oxidized sulfide only with nitrate as the sole electron acceptor, while the latter only oxidized sulfide with N_2_O (Sorokin, 2003; Boltianskaia et al., 2007). A similar trait was also observed in some autotrophic strains such as *Thioalkalivibrio* and *Alkalispirallum* (Sorokin et al., 2001, 2006). The oxidation of sulfide combined with the reduction of nitrogen compounds indicated the possible coupling of the nitrogen and sulfur cycles in the natural environment.

### Nitrogen Metabolism of the Genus *Halomonas*

#### The Co-occurrence of Denitrification Pathways in the “*Halomonas desiderata* Group”

Bioinformatic analysis showed that all 14 genomes in this study possessed key genes for the complete denitrification pathways, including both the membrane-bound respiratory nitrate reductase NAR (*narG*) and the periplasmic nitrate reductase NAP (*napA*), cytochrome cd1-type nitrite reductase NIR (*nirS*), cytochrome *c* nitric oxide reductase NOR (*norBC*), and nitrous oxide reductase NOS (*nosZ*). Nearly the same co-occurrence patterns of these denitrification genes were observed in these 14 strains, as well as in other strains belonging to the “*H. desiderata* group,” except for *H. saliphila* LCB169^T^ (Figure 2).

One feature of the denitrification pathways of the “*H. desiderata* group” was the coexistence of two types of dissimilatory nitrate reductases, NAR and NAP, in one organism. Within this group, only two strains, *H. endophytica* MC28^T^ and *Halomonas* sp. DX6, lack NAP. In general, the membrane-bound NAR is responsible for anaerobic nitrate respiration, while the periplasmic NAP can reduce nitrate irrespective of O_2_ (Richardson et al., 2001; Sparacino-Watkins et al., 2014). In the “*H. desiderata* group,” *nap* operons were distantly located in genomes, far away from other denitrification gene clusters. On the contrary, *narGHJI* operons were located within a large chromosomal region that encodes all the other genes involved in the denitrification pathway, including *nirS*, *nor*, and *nos* operons, as well as transporters and transcriptional regulators. Upstream of *nar* operons was the *nor* gene cluster *norECBQD*. The *nirS1* gene, which encodes the catalytic subunit of nitrite reductase, was present upstream of the *nosRZDFYL* operon. At least three nitrate/nitrite transporter-encoding genes were also located in this region. All of these strains can reduce nitrate and nitrite under aerobic and anaerobic conditions, with N_2_O and N_2_ as the gaseous products of denitrification, consistent with the existence of *nar*, *nap*, *nir*, *nor*, and *nos* operons.

Multiple transcriptional regulators have also been identified nearby the denitrification gene clusters in the “*H. desiderata* group,” implying their possible roles in the regulation of denitrification. *narX-narL*, which encodes a two-component system, NarXL, acting as a sensor and a regulator in response to nitrate and/or nitrite (Hartig et al., 1999), was located upstream of *narGHJI*. This two-component system was found to be closely linked with the *nar* operon. In addition, at least three genes encoding Crp/Fnr family transcription factors, which have been recognized as major players in the denitrification regulatory network (Spiro, 1994), were also found nearby denitrification gene clusters. One *fnr1* gene, encoding an FNR-type regulator, was located downstream of *nor* operons. Two other genes, *dnr1* and *dnr2*, encoding FNR-like regulators affiliated with the Dnr subgroup of the Crp/Fnr family, were also identified. Among them, *dnr1* was found adjacent to *nirS1*, while *dnr2* lied downstream of *narGHJI* operons. It is interesting to note that *dnr1* was almost exclusively present in the “*H. desiderata* group” strains, showing a significant positive correlation with *nirS1*, implying a possible unique mechanism of *nirS1* regulated by *dnr1* in the “*H. desiderata* group.” Moreover, nearly all the denitrifiers in the genus *Halomonas* possessed *dnr2*, except for *Halomonas litopenaei* and *Halomonas titanicae*, implying its possible role in the regulation of the denitrification process. Other genes encoding two known dedicated NO sensors, NsrR and NorR (Bodenmiller and Spiro, 2006; Bush et al., 2011), as well as NnrS, which is involved in defense against NO in nitrosative stress (Stern et al., 2013), were present close to the *nos* gene cluster, while another *nnrS* gene was located close to the *nir* operon, indicating a unique sensitive mechanism of NO detoxification. These sensors and regulators might sense different environmental signals and be involved in the complex regulatory network of the denitrification pathway in the “*H. desiderata* group.”

#### The Distinct Evolutionary Lineage of Nitrite Reductase in the “*Halomonas desiderata* Group”

The “*H. desiderata* group” formed a distinct clade in the phylogeny of the nitrite reductase NirS, similar to their phylogenomic relationships. Phylogenetic analysis of NirS sequences from the genus *Halomonas* confirmed the presence of two distinct evolutionary clades, clade I and II, represented by NirS1 and NirS2, respectively (Supplementary Figure 5). Clade I was mainly composed of NirS1-type sequences from nearly all members of the “*H. desiderata* group,” plus *Halomonas alkalicola*, *Halomonas daqiaonensis*, and *Halomonas campaniensis*, and grouped with sequences from diverse taxonomic members of Gammaproteobacteria and Alphaproteobacteria. Clade II harbored NirS2-type sequences from the remaining denitrifying species of the genus *Halomonas* and from *Marinobacter* and *Pseudomonas* species. Amino acid sequence alignment showed that NirS1-type sequences obtained from the “*H. desiderata* group” shared only 36–46% identities with NirS2-type sequences of the genus *Halomonas*. The NirS phylogeny implied that the two clades underwent different evolutionary processes, suggesting that *nirS1* might have been horizontally transferred to the “*H. desiderata* group,” while *nirS2* was present due to vertical transfer. The *nirS* gene is usually found to cluster with ancillary genes, as commonly described for other denitrifiers, such as those from *Pseudomonas* and *Marinobacter* (Bali et al., 2014). However, in the “*H. desiderata* group,” the *nirS1* genes were located upstream from the *nos* operon, far away from the ancillary genes *nirCFDLGHJEN*, which are essential for electron transfer and maturation of cytochrome *cd1* nitrite reductase, whereas the *nirS2* gene clustered with ancillary genes (*nirCFSDLGHJEN*) and was located neighboring with the *norBC* operon. Obviously, the gene locus of *nirS1* in the genome was different from that of *nirS2*.

#### The Co-occurrence of Different Types of Nitrite Reductase in the “*Halomonas desiderata* Group”

The occurrence patterns of nitrite reductase genes differed greatly among the denitrifiers in the “*H. desiderata* group,” depending on whether two divergent *nirS* genes coexist or not and the co-occurrence of *nirS* and *nirK*. It is worth noting that three strains in the “*H. desiderata* group,” including MCCC 1A11036^T^, *Halomonas* sp. D167-6-1, and *H. azerbaijanica* TBZ202^T^, harbored two divergent copies of *nirS* (*nirS1* and *nirS2*). Previously, the co-occurrence of two divergent *nirS* genes in one organism was reported in a denitrifying *Thauera* sp. strain 27, in which one *nirS* gene was positively regulated by nitrate while the other was constitutively expressed, suggesting an increased competitive ability under different conditions (Etchebehere and Tiedje, 2005). Moreover, the coexistence of the two evolutionary unrelated variants *NirK* and *NirS* in one organism was also observed in four genomes, including MCCC 1A11059^T^, MCCC 1A17488, *H. lactosivorans* KCTC 52281^T^, and *H. saliphila* L5. The *nirK* gene in the genomes was found as a single gene, not within a larger operon, and far away from other denitrification gene clusters. It has been long considered that both types of Nir are mutually exclusive in one organism (Jones et al., 2008), and communities of denitrifiers with NirS responded more flexibly to environmental gradients than those with NirK, which suggests that these communities occupy different ecological niches (Jones and Hallin, 2010). A recent survey of genomes revealed that a small number of organisms harbor both genes in their genomes (Graf et al., 2014). The co-occurring *nirS* and *nirK* in *Bradyrhizobium oligotrophicum* strain S58 were found to be functionally redundant, while the two types of Nir in *Pseudomonas stutzeri* strain JM300 were functionally different (Sanchez and Minamisawa, 2018; Wittorf et al., 2018). The presence of two types of Nir or multiple copies of *nirS* in the same organism is likely to be related with their metabolic needs. Whether these enzymes are truly functional when present in one organism in the genus *Halomonas* is unclear.

#### Co-occurrence Patterns of Denitrification Genes in the Genus *Halomonas*

In order to further understand the denitrification potential within the genus *Halomonas*, a comprehensive comparative analysis was conducted based on 206 *Halomonas* genomes. The inventory of denitrification genes varied in all these 206 genomes (Supplementary Figure 1 and Supplementary Table 5). Most bacteria (158/206) possess at least one copy of *narGHJI*, implying the importance of respiratory nitrate reduction for *Halomonas.* Besides, 53 of 206 genomes carried at least one copy of *napA*. The coexistence of NAR and NAP in one organism was present in 42 genomes, mostly belonging to the “*H. desiderata* group” (30/42). They may function in denitrification or dissimilatory nitrate reduction to ammonium (DNRA). Upon inspection of 206 genomes of *Halomonas*, 66 had *nirS*, but only 10 possessed *nirK*, indicating that this genus should be NirS-type denitrifiers. The common occurrence of cytochrome cd1-type nitrite reductase in denitrifiers of *Halomonas* suggests that this genus might have inherited *nirS* genes from a common ancestor, and later some species acquired *nirK*.

Except for the “*H. desiderata* group,” there are several other groups of denitrifying bacteria within the genus *Halomonas* that also possess complete or truncated denitrification pathways. Co-occurrence of *narG*, *nirS2*, *c-nor*, and *nosZ* genes and the lack of a *nap* gene were found in at least 20 other denitrifying species of *Halomonas*, including *Halomonas denitrificans*, *Halomonas halodenitrificans*, and *H. litopenaei*. Notably, a majority of these denitrifiers prefer NirS2-type nitrite reductases instead of NirS1, in contrast to the “*H. desiderata* group,” which prefers NirS1. With respect to nitric oxide reductases, at least five species, including *Halomonas xinjiangensis*, *Halomonas radicis*, *Halomonas lutea*, *Halomonas xianhensis*, and *Halomonas ilicicola*, harbored qNor instead of *c*-Nor. Besides, a truncated denitrification pathway was present in a phylogenetically closely related clade designated as the “*H. titanicae* group,” including at least four species (*H. titanicae*, *Halomonas olivaria*, *Halomonas neptunia*, and *Halomonas alkaliantarctica*), which were found dominant in deep sea environments (Kaye et al., 2011; Salazar et al., 2016). Nineteen genomes in this group exhibited incomplete pathways, with the existence of *nar*, *c-Nor*, and *nos* operons. However, *nirS* and *nirK*, which are hallmark genes of the denitrification process, were not identified in the genomes of this group, suggesting that this process of NO_2_^–^ reduction to NO is not carried out, other enzymes play this role, or even chemodenitrification takes place (Onley et al., 2018).

Phylogenies of denitrification functional genes including *nirS*, *napA*, *narG*, *norB*, and *nosZ* showed diverse evolutionary clades in the genus *Halomonas* (Supplementary Figures 5–9). Incongruence of the phylogenies was observed when comparing these functional genes with 16S rRNA gene (Supplementary Figure 10), except for the *narG* genes, which suggested that the denitrification genes in the genus *Halomonas* may have undergone different evolutionary processes. According to Petri and Imhoff, respiratory nitrate reductase genes went through a long evolutionary history and already played a key role in energy metabolism during peroxic times (Petri and Imhoff, 2000). Delorme et al. hypothesized that the acquisition of the ability to reduce other nitrogen oxides was probably a more recent event than that of nitrate reduction (Delorme et al., 2003). The diverse co-occurrence patterns as well as different evolutionary lineages of denitrification genes distributed among different phylogenetic clades of the genus *Halomonas* indicated that they might be involved in the nitrogen cycle in distinct ecological niches.

#### Widespread Presence of the Inorganic Nitrogen Assimilation Pathway Within the Genus *Halomonas*

Genomic analyses further revealed complete nitrate and nitrite assimilation pathways in the genus *Halomonas*. Assimilatory nitrate reduction gene clusters (*nas*) encoding NADH-nitrite oxidoreductase (*nirBD*) and assimilatory nitrate reductase (*nasA*) were annotated in these genomes, allowing the production of ammonia for L-glutamate biosynthesis and implying the ability of growth under nitrate or nitrite as the sole nitrogen source. Putative regulatory proteins involved in nitrate assimilation were identified in these genomes, such as the transcription antiterminator nasR and the two-component regulatory system NasS–NasT (Luque-Almagro et al., 2011). NasST homologues are common in the “*H. desiderata* group” strains, while nasR homologues are present in 15 of them, represented by MCCC 1A14433^T^, MCCC 1A11081^T^, and MCCC 1A11058^T^, implicating possible dual regulation. However, the occurrence of NasR and NasST was variable, depending on the organism, and seemed almost mutually exclusive, as distributed in the two major phylogenetic clades within the genus *Halomonas*, as shown in Figure 2 and Supplementary Figure 1. Although the nasR homologues from these genomes showed low amino acid identities (about 30%) with that from *Klebsiella pneumoniae* M5al (Goldman et al., 1994), they were composed of an N-terminal NO_3_^–^/NO_2_^–^-sensing NIT domain and a C-terminal ANTAR signaling domain, indicating their potential role in the regulation of nitrate assimilation. Besides, the ubiquitous pathways for ammonium assimilation and regulation were also widespread in the genus *Halomonas*. In addition to inorganic nitrogen, urea seemed to be an alternative nitrogen source, due to the presence of the *urtABCDE* and *ureDABCEFGJ* gene clusters, which encode the urea ABC transporter and urease, respectively. They might utilize nitroalkane and nitrile based on the presence of nitronate monooxygenase and nitrilase. Their ability to utilize various nitrogen sources would facilitate their adaptation to varied habitats.

### Sulfur Oxidation Pathway

#### Sulfur Oxidation Pathways in the 14 Strains

In this report, all 14 strains were capable of oxidizing sulfide aerobically. The sulfide oxidization product was thiosulfate instead of sulfite. Correspondingly, the genes responsible for sulfide oxidation were inspected in these strains and compared with other species of the genus *Halomonas* (Figure 2 and Supplementary Table 5). As a result, *fccAB* genes encoding flavocytochrome *c* sulfide dehydrogenase (FCSD) were identified in all the 14 strains, while the sulfide:quinone oxidoreductase (SQR) gene *sqr* co-occurred with *fccAB* genes in eight strains, i.e., MCCC 1A05748, MCCC 1A05775, MCCC 1A11059^T^, MCCC 1A11062, MCCC 1A13718^T^, MCCC 1A17486, MCCC 1A17488, and MCCC 1A17499. The simultaneous presence of *fccAB* and *sqr* genes was also observed in nine other genomes, such as those of *H. campisalis* MCCC 1A11675^T^, *H. daqingensis* DQD2-30^T^, *H. montanilacus* PYC7W^T^, *H. endophytica* MC28^T^, *H. kenyensis* AIR-2^T^, *H. alkalicola* JS92-SW72, and *Halomonas pantelleriensis* AAP^T^. Upon inspection of 206 genomes in the genus *Halomonas*, only 38 genomes contained *fccAB* gene, and most of them (33/38) belonged to the “*H. desiderata* group.” On the contrary, *sqr* genes were widely distributed in diverse species through the genus *Halomonas* (Supplementary Figures 11, 12).

In addition, the persulfide dioxygenase gene *pdo* was commonly present in the genus *Halomonas*, including the 14 genomes reported here. Heterotrophic bacteria containing *sqr*/*fccAB* and *pdo* genes can oxidize sulfide to sulfite and thiosulfate, which plays a possible role in sulfide detoxification (Xia et al., 2017). The coexistence of *sqr/fccAB* and *pdo* in these 14 strains would well explain their ability of sulfide oxidation. SQR is thought to be widespread among prokaryotes and appears to be critical for sulfide oxidation (Reinartz et al., 1998), while FccAB is thought to be more prevalent at low sulfide concentrations (Mussmann et al., 2007). In this study, the eight strains harboring *sqr* exhibited stronger sulfide oxidation than *fccAB*-carrying strains, especially strains MCCC 1A11059^T^, MCCC 1A17488, and MCCC 1A13718^T^. These putative SQR sequences shared 88.4–100% similarities with each other and grouped together. Although they showed only about 35% sequence identities with that from *Pseudomonas putida* (UniParc ID: UPI000000E9AA), they grouped with Type II SQRs in the phylogenetic tree and shared conserved amino acid sequences with the biochemically well-characterized SQR from *Aquifex aeolicus* (UniParc ID: UPI00000567FF) (Sousa et al., 2018; Supplementary Figures 12,13).

In addition to sulfide oxidation, the three strains, MCCC 1A11059^T^, MCCC 1A17488, and MCCC 1A13718^T^, can also convert thiosulfate to tetrathionate. Accordingly, the thiosulfate dehydrogenase-encoding gene *tsdA* was identified in these three strains (Supplementary Figure 14). Their *tsdA*-like gene was accompanied by the *tsdB* gene, encoding a diheme cytochrome *c*. Although these TsdA homologues showed low amino acid sequence identities (only about 35%) with functionally verified TsdA of *Allochromatium vinosum* (Brito et al., 2015), they contained the characteristic heme groups and conserved amino acid residues, which are essential for the catalytic activity of thiosulfate dehydrogenase (Denkmann et al., 2012; Supplementary Figure 15). TsdA homologues are widespread among prokaryotes, not only in specialized sulfur oxidizers but also in many chemoorganoheterotrophs. The occurrence of *tsdA*-related genes in *Halomonas* strains is in agreement with their ability of tetrathionate formation, as they might oxidize thiosulfate as supplemental energy just like in *Pseudomonas* spp. (Denkmann et al., 2012).

#### Widespread Presence of Sulfur Oxidation Pathways in the Whole Genus *Halomonas*

The occurrence pattern of the sulfur oxidation pathways was further compared within the genus *Halomonas* (Supplementary Figure 1 and Supplementary Table 5). In the 206 genomes searched, 65 genomes of diverse species contained at least one copy of the *sqr* gene, while four harbored two divergent copies of *sqr*. SQRs were widespread throughout the genus *Halomonas*, while FCSD was almost exclusively found in the “*H. desiderata* group.” The phylogenetic analysis showed that the SQR homologues retrieved from the genus *Halomonas* were very diverse and divided into at least three different groups of SQRs (Types II, III, and V), according to a previous SQR classification system (Marcia et al., 2010; Sousa et al., 2018; Supplementary Figure 12). A majority of these SQR homologues obtained from *Halomonas* belonged to Type II SQRs, forming at least three clades in the dendrogram. Clade I showed high amino acid sequence identities with that of *P. putida* (UniParc ID: UPI000000E9AA) ranging from 57 to 63%, clade II showed 47–50% sequence similarity, and clade III 35–38%. In addition, three sequences obtained from *H. denitrificans* DSM 18045^T^, *Halomonas nitroreducens* 11S^T^, and *Halomonas* sp. ES 049 grouped with Type III SQRs, while one from *H. alkaliantarctica* SAT111 belonged to Type V. The SQR homologues from *Halomonas* were disperse in the dendrogram, pointing to possible lateral gene transfer events. Some *sqr*-containing heterotrophic bacteria are reported to harbor *sqr* and *pdo* in a gene cluster (Xia et al., 2017); however, the *sqr* and *pdo* genes were separate in most *Halomonas* genomes, with the exception of clade I *sqr*, which was located adjacent to *pdo* in the genome.

Besides, almost all 206 of the genomes possessed *pdo* genes (204/206), 14 of which contained two or three copies of *pdo*. PDO homologues retrieved in this study showed more than 50% amino acid sequence identities with those from *Pseudomonas aeruginosa* (NP_251605) and *P. putida* (ABQ76243) and belonged to Group II PDOs (Supplementary Figure 16). Co-occurrence of SQR/FCSD (or both) and PDO is observed in 81 genomes, indicating that it is quite common in *Halomonas* spp. to oxidize sulfide to sulfite and thiosulfate. Likewise, TsdA homologues are also ubiquitous in most species of *Halomonas* (106/206), suggesting the ability of thiosulfate oxidation of this genus. Coexistence of SQR/FCSD, PDO, and TsdA was observed in 37 genomes, distributed in more than 20 *Halomonas* species, such as *Halomonas aestuarii*, *H. denitrificans*, *Halomonas fontilapidosi*, *Halomonas jeotgali*, *H. halodenitrificans*, *H. nitroreducens*, *Halomonas boliviensis*, and *H. titanicae*, in addition to MCCC 1A13718^T^, MCCC 1A11059^T^, and MCCC 1A17488, most of which are denitrifying species, indicating that they might be able to remove sulfide, thiosulfate, and nitrate simultaneously. Considering their excellent removal of sulfide and nitrate, *Halomonas* bacteria might have promising applications in the biotechnology industry.

Sulfide oxidation by heterotrophic denitrifiers has been previously reported in *H. mongoliensis* and *H. kenyensis*, as well as several other *Halomonas* species (Sorokin, 2003; Boltianskaia et al., 2007). In a continuous integrated autotrophic-heterotrophic denitrification bioreactor, *H. desiderata* was observed to dominate, showing the ability to simultaneously remove sulfide and nitrate (Zhang et al., 2020). *Halomonas* was also found to dominate; it could enhance sulfide denitrification removal on high-salinity wastewaters (Liu et al., 2016a). Besides, bacterial thiosulfate oxidation was also found in *Halomonas* isolates from deep sea sediment (Teske et al., 2000; Du et al., 2019). This is the first time the sulfur oxidation pathways in the genus *Halomonas* are depicted by retrieving their functional genes. Our results support that bacteria of *Halomonas* having sulfur oxidation potential are more common than previously recognized, implying their ecological contribution to the sulfur cycle.

### Other Metabolic Pathways

#### Carbon Metabolism

The heterotrophic lifestyle of the 14 strains was inferred from the existence of organic carbon degradation pathways but lacks key genes for carbon fixation. As predicted from annotation results, all CO_2_ fixation pathways were absent in these six novel species and other *Halomonas* genomes used in this study, except for *Halomonas heilongjiangensis* (with the rTCA cycle and the Calvin cycle) and *Halomonas anticariensis* (with the Calvin cycle).

#### Polyhydroxyalkanoate and Exopolysaccharide Synthesis

Polyhydroxyalkanoates can serve as carbon and energy reserves and can be naturally synthesized by numerous bacterial species. Genes coding for the key enzymes involved in PHA synthesis pathway, including acetyl-CoA acetyltransferase (PhaA), acetoacetyl-CoA reductase (PhaB), poly-β-hydroxybutyrate polymerase (PhaC), and PHA synthesis repressor (PhaR), which belonged to the class I PHA synthases, were identified in all 14 of these genomes as well as other *Halomonas* genomes (Supplementary Figure 1 and Supplementary Table 5). The PHA biosynthesis genes *phaA*, *phaB*, *phaC*, and *phaR* were common in the genus *Halomonas*, and did not cluster in one operon but were distant from each other. This kind of organization was obviously different from that of typical PHA synthase genes, which are usually organized in one operon. Besides, various putative EPS biosynthesis and export gene clusters were identified in these genomes, such as *pgaABC* (encoding biofilm PGA synthesis protein), *lapAB* (encoding lipopolysaccharide assembly protein), *lptABC* (encoding the lipopolysaccharide export system), and genes encoding polysaccharide biosynthesis proteins, and the polysaccharide export protein Wza, implying the ability to produce EPS.

#### Phosphonate Metabolism

Gene clusters coding for complete phosphonate transport and metabolism pathways, including *phnCDEFG* (encoding the phosphonate ABC transporter and regulator) and *phnHIJKLMNP* [encoding a carbon-phosphorus (CP) lyase], were only present in MCCC 1A11036^T^, MCCC 1A14433^T^, and MCCC 1A11057 and were absent in 11 other strains (Supplementary Figure 1 and Supplementary Table 5), suggesting that these strains might metabolize ubiquitous phosphonate by CP lyase as a phosphorus or carbon source.

#### Compatible Solute Biosynthesis

A three-gene cluster (*ectA*, *ectB*, and *ectC*) responsible for the synthesis of ectoine (1,4,5,6-tetrahydro-2-methyl-4-pyrimidinecarboxylic acid), a common osmoprotectant in many halophilic eubacteria (Schwibbert et al., 2011), was identified in these 207 genomes. Other organic osmotic protectants, such as glycine betaine, may also be synthesized via choline dehydrogenase (BetA) and betaine aldehyde dehydrogenase (BetB), similar to *Halomonas elongata* (Cánovas et al., 2000). The presence of these genes was common in the genus *Halomonas* (Supplementary Table 5), which might explain the adaptation of *Halomonas* to high salinity.

#### Heavy Metal Resistance-Related Genes

These genomes carry genes encoding copper resistance proteins and transporters, implying the tolerance to such bio-toxic metals in the environment. Besides, multiple genes potentially involved in arsenic resistance are identified in these genomes, including two arsenic resistance operons containing genes encoding ArsH and ACR3 and other related genes encoding the arsenical resistance operon repressor ArsR, as well as the arsenite-transporting ATPase ArsA. However, proteins homologous to aerobic arsenite oxidase (AioA and AioB) and anaerobic arsenite oxidase (ArxA) (Lin et al., 2012; Hamamura et al., 2014) could not be identified in these genomes, although they were found in several closely related species (*H. campisalis* and *H. montanilacus*), as well as other species, such as *H. aestuarii*, *Halomonas andesensis*, and *H. boliviensis*, as shown in Supplementary Figure 1 and Supplementary Table 5.

## Taxonomy

### Description of *Halomonas zhangzhouensis* sp. nov.

*Halomonas zhangzhouensis* (zhang.zhou.en’sis. N.L. fem. adj. *zhangzhouensis*, of or pertaining to Zhangzhou, a city in Fujian, China, where the type strain was isolated).

Cells are Gram-stain-negative, facultatively anaerobic, motile by means of peritrichous flagella, rod-shaped, 0.5–0.7 μm in width, and 1.0–3.0 μm in length. Colonies are yellow, circular, smooth, and opaque on MA after 72 h at 32°C. Growth occurs at 4–50°C (optimum, 37–40°C) and in pH 7–10 (optimum, 7–8). The NaCl concentration range for growth is 0–18% (optimum, 2–8%, w/v). Catalase and oxidase are positive. N_2_ and N_2_O are the major gaseous products of aerobic and anaerobic denitrification. Bacteria are weak positive for the Voges–Proskauer test and fermentation of D-glucose and negative for the ONPG test, indole production, H_2_S production, and hydrolysis of Tween-20, -40, -60, -80, gelatin, cellulose, starch, casein, and DNA. In the API ZYM tests, bacteria are positive for alkaline phosphatase, esterase, valine aminopeptidase, acid phosphatase, naphthol-AS-Bl-phosphoamidase, esterase lipase (C8), lipase (C14), leucine aminopeptidase, and cystine aminopeptidase and negative for α-chymotrypsin, α-galactosidase, β-galactosidase, β-glucuronidase, β-glucosidase, *N*-acetyl-β-glucosaminidase, α-mannosidase, α-fucosidase, trypsin, and α-glucosidase. In the API 20NE tests, bacteria are positive for utilization of L-arabinose, D-mannitol, potassium gluconate, malic acid, and trisodium citrate and negative for arginine dihydrolase, β-glucosidase, lysine decarboxylase, ornithine decarboxylase, urease, tryptophan deaminase, and utilization of D-glucose, D-mannose, *N*-acetyl-glucosamine, D-maltose, capric acid, adipic acid, and phenylacetic acid. The predominant respiratory quinone is Q-9. The principal cellular fatty acids are summed feature 8 (C_18:1_ *ω*7*c*), C_16:0_, and C_19:0_ cyclo *ω*8*c*. The polar lipids are DPG, PG, PE, PL, and six unknown polar lipids. The genomic G+C content of the type strain is 63.3%.

The type strain CXT3-11^T^ ( = MCCC 1A11036^T^ = KCTC 72087^T^) was isolated from sediments of a shrimp culture pond, Zhangzhou City, China.

### Description of *Halomonas aerodenitrificans* sp. nov.

*Halomonas aerodenitrificans* (a.e.ro.de.ni.tri’fi.cans. Gr. masc. n. *aer*, *aeros*, air; N.L. v. *denitrificare*, to denitrify; N.L. part. adj. *aerodenitrificans*, denitrifying with or in air).

Cells are Gram-stain-negative, facultatively anaerobic, motile by means of peritrichous flagella, rod-shaped, 0.6–1.1 μm in width, and 1.7–2.7 μm in length. Colonies are yellow, circular, smooth, and opaque on MA after 72 h at 32°C. Growth occurs at 4–50°C (optimum, 37°C) and in pH 7–10 (optimum, 7–8). The NaCl concentration range for growth is 0–18% (optimum, 2–8%, w/v). Catalase and oxidase are positive. N_2_ and N_2_O are the major gaseous products of aerobic and anaerobic denitrification. Bacteria are positive for the Voges–Proskauer test, fermentation of D-glucose, and hydrolysis of Tween-20, -40, -60, -80, and starch and negative for the ONPG test, indole production, H_2_S production, and hydrolysis of gelatin, cellulose, casein, and DNA. In the API ZYM tests, bacteria are positive for alkaline phosphatase, esterase, valine aminopeptidase, acid phosphatase, naphthol-AS-Bl-phosphoamidase, esterase lipase (C8), lipase (C14), leucine aminopeptidase, cystine aminopeptidase, trypsin, and α-glucosidase and negative for α-chymotrypsin, α-galactosidase, β-galactosidase, β-glucuronidase, β-glucosidase, *N*-acetyl-β-glucosaminidase, α-mannosidase, and α-fucosidase. In the API 20NE tests, bacteria are positive for utilization of D-glucose, D-mannitol, D-maltose, potassium gluconate, adipic acid, malic acid, and trisodium citrate and negative for arginine dihydrolase, β-glucosidase, gelatinase, lysine decarboxylase, ornithine decarboxylase, urease, tryptophan deaminase, and utilization of L-arabinose, D-mannose, *N*-acetyl-glucosamine, capric acid, and phenylacetic acid. The predominant respiratory quinone is Q-9. The principal cellular fatty acids are summed feature 8 (C_18:1_ *ω*7*c*) and C_16:0_. The polar lipids are DPG, PG, PE, four PLs, and five unknown polar lipids. The genomic G+C content of the type strain is 64%.

The type strain CYD-9^T^ ( = MCCC 1A11058^T^ = KCTC 72088^T^) was isolated from coastal water from the Taiwan Strait, China.

### Description of *Halomonas sulfidoxydans* sp. nov.

*Halomonas sulfidoxydans* (sul.fid.o’xy.dans. N.L. neut. n. *sulfidum*, sulfide; N.L. part. adj. *oxydans*, oxidizing; N.L. part. adj. *sulfidoxydans*, oxidizing sulfides).

Cells are Gram-stain-negative, facultatively anaerobic, motile by means of peritrichous flagella, rod-shaped, 0.7–0.8 μm in width, and 1.3–2.0 μm in length. Colonies are yellow, circular, smooth, and opaque on MA after 72 h at 32°C. Growth occurs at 4–50°C (optimum, 37–40°C) and in pH 6–10 (optimum, 7–8). The NaCl concentration range for growth is 0–18% (optimum, 2–6%, w/v). Catalase and oxidase are positive. N_2_ and N_2_O are the major gaseous products of aerobic and anaerobic denitrification. Bacteria are positive for the Voges–Proskauer test, fermentation of D-glucose, and hydrolysis of gelatin and negative for the ONPG test, indole production, H_2_S production, and hydrolysis of Tween-20, -40, -60, -80, starch, cellulose, casein, and DNA. In the API ZYM tests, bacteria are positive for alkaline phosphatase, esterase, valine aminopeptidase, acid phosphatase, naphthol-AS-Bl-phosphoamidase, esterase lipase (C8), lipase (C14), leucine aminopeptidase, cystine aminopeptidase, trypsin, and α-glucosidase and negative for α-chymotrypsin, α-galactosidase, β-galactosidase, β-glucuronidase, β-glucosidase, *N*-acetyl-β-glucosaminidase, α-mannosidase, and α-fucosidase. In the API 20NE tests, bacteria are positive for arginine dihydrolase, gelatinase, and utilization of D-glucose, L-arabinose, D-mannitol, D-maltose, potassium gluconate, adipic acid, malic acid, and trisodium citrate and negative for β-glucosidase, lysine decarboxylase, ornithine decarboxylase, urease, tryptophan deaminase, utilization of D-mannose, *N*-acetyl-glucosamine, capric acid and phenylacetic acid. The predominant respiratory quinone is Q-9. The principal cellular fatty acids are summed feature 8 (C_18:1_ *ω*7*c*), summed feature 3 (C_16:1_ *ω*7*c* and/or C_16:1_ *ω*6*c*), and C_16:0_. The polar lipids are DPG, PG, PE, PC, AL, APL, and two PLs. The genomic G+C content of the type strain is 66%.

The type strain CYN-1-2^T^ ( = MCCC 1A11059^T^ = KCTC 72089^T^) was isolated from coastal surface sediments of the Taiwan Strait, China.

### Description of *Halomonas ethanolica* sp. nov.

*Halomonas ethanolica* (e.tha.no’li.ca N.L. neut. n. *ethanol*, ethanol; L. fem. suff. -*ica*, suffix used with various meanings; N.L. fem. adj. *ethanolica*, belonging to ethanol, in reference to the ability of the species to utilize ethanol as a substrate for growth).

Cells are Gram-stain-negative, facultatively anaerobic, motile by means of peritrichous flagella, rod-shaped, 0.6–0.8 μm in width, and 1.6–2.4 μm in length. Colonies are yellow, circular, smooth, and opaque on MA after 72 h at 32°C. Growth occurs at 4–50°C (optimum, 37–40°C) and in pH 7–10 (optimum, 7–8). The NaCl concentrations range for growth is 0–18% (optimum, 1–10%, w/v). Catalase and oxidase are positive. N_2_ and N_2_O are the major gaseous products of aerobic and anaerobic denitrification. Bacteria are positive for the Voges–Proskauer test, fermentation of D-glucose, and hydrolysis of Tween-20, -40, -60, and -80 and negative for the ONPG test, indole production, H_2_S production, and hydrolysis of starch, cellulose, gelatin, casein, and DNA. In the API ZYM tests, bacteria are positive for alkaline phosphatase, esterase, valine aminopeptidase, acid phosphatase, naphthol-AS-Bl-phosphoamidase, esterase lipase (C8), leucine aminopeptidase, cystine aminopeptidase, and α-glucosidase and negative for α-chymotrypsin, α-galactosidase, β-galactosidase, β-glucuronidase, β-glucosidase, lipase (C14), trypsin, *N*-acetyl-β-glucosaminidase, α-mannosidase, and α-fucosidase. In the API 20NE tests, bacteria are positive for utilization of D-glucose, D-mannitol, D-maltose, potassium gluconate, adipic acid, malic acid, and trisodium citrate and negative for arginine dihydrolase, β-glucosidase, gelatinase, lysine decarboxylase, ornithine decarboxylase, urease, tryptophan deaminase, and utilization of L-arabinose, D-mannose, *N*-acetyl-glucosamine, capric acid, and phenylacetic acid. The predominant respiratory quinone is Q-9. The principal cellular fatty acids are summed feature 8 (C_18:1_ *ω*7*c*) and C_16:0_. The polar lipids are DPG, PG, PE, and PL. The genomic G+C content of the type strain is 64.5%.

The type strain CYT3-1-1^T^ ( = MCCC 1A11081^T^ = KCTC 72090^T^) was isolated from sediments of a shrimp culture pond, Zhangzhou City, China.

### Description of *Halomonas sulfidivorans* sp. nov.

*Halomonas sulfidivorans* (sul.fi.di.vo’rans. N.L. neut. n. *sulfidum*, sulfide; L. pres. part. *vorans*, devouring; N.L. part. adj. *sulfidivorans*, sulfide-devouring).

Cells are Gram-stain-negative, facultatively anaerobic, motile by means of peritrichous flagella, rod-shaped, 0.6–0.7 μm in width, and 1.7–2.4 μm in length. Colonies are yellow, circular, smooth, and opaque on MA after 72 h at 32°C. Growth occurs at 4–55°C (optimum, 37–40°C) and in pH 6–10 (optimum, 7–8). The NaCl concentration range for growth is 0–20% (optimum, 2–8%, w/v). Catalase and oxidase are positive. N_2_ and N_2_O are the major gaseous products of aerobic and anaerobic denitrification. Bacteria are positive for the Voges–Proskauer test, fermentation of D-glucose, and hydrolysis of starch and DNA and negative for the ONPG test, indole production, H_2_S production, hydrolysis of Tween-20, -40, -60, -80, gelatin, cellulose, and casein. In the API ZYM tests, bacteria are positive for alkaline phosphatase, esterase, valine aminopeptidase, acid phosphatase, naphthol-AS-Bl-phosphoamidase, esterase lipase (C8), lipase (C14), leucine aminopeptidase, and cystine aminopeptidase and negative for α-chymotrypsin, α-galactosidase, β-galactosidase, β-glucuronidase, β-glucosidase, *N*-acetyl-β-glucosaminidase, α-mannosidase, α-fucosidase, trypsin, and α-glucosidase. In the API 20NE tests, bacteria are positive for utilization of D-glucose, L-arabinose, D-mannitol, *N*-acetyl-glucosamine, D-maltose, potassium gluconate, adipic acid, malic acid, and trisodium citrate and negative for arginine dihydrolase, β-glucosidase, gelatinase, lysine decarboxylase, ornithine decarboxylase, urease, tryptophan deaminase, and utilization of D-mannose, capric acid, and phenylacetic acid. The predominant respiratory quinone is Q-9. The principal cellular fatty acids are summed feature 8 (C_18:1_ *ω*7*c*), C_16:0_, summed feature 3 (C_16:1_ *ω*7*c*/C_16:1_ *ω*6*c*), and C_12:0_ 3-OH. The polar lipids are DPG, PG, PE, AL, PL, and five unknown polar lipids. The genomic G+C content of the type strain is 64.2%.

The type strain NLG_F1E^T^ ( = MCCC 1A13718^T^ = KCTC 72091^T^) was isolated from deep sea sediments, Pacific Ocean.

### Description of *Halomonas tianxiuensis* sp. nov.

*Halomonas tianxiuensis* (tian.xiu.en’sis. N.L. fem. adj. *tianxiuensis*, pertaining to the Tianxiu Hydrothermal Field, on the Northwest Indian Ridge, from where the type strain was isolated).

Cells are Gram-stain-negative, facultatively anaerobic, motile by means of peritrichous flagella, rod-shaped, 0.6–0.7 μm in width, and 1.3–2.1 μm in length. Colonies are yellow, circular, smooth, and opaque on MA after 72 h at 32°C. Growth occurs at 4–50°C (optimum, 37–40°C) and in pH 6–10 (optimum, 7–8). The NaCl concentration range for growth is 0–18% (optimum, 2–8%, w/v). Catalase and oxidase are positive. N_2_ and N_2_O are the major gaseous products of aerobic and anaerobic denitrification. Bacteria are positive for the Voges–Proskauer test, fermentation of D-glucose, and hydrolysis of starch and DNA and negative for the ONPG test, indole production, H_2_S production, and hydrolysis of Tween-20, -40, -60, -80, gelatin, cellulose, and casein. In the API ZYM tests, bacteria are positive for alkaline phosphatase, esterase, valine aminopeptidase, acid phosphatase, naphthol-AS-Bl-phosphoamidase, lipase (C14), leucine aminopeptidase, cystine aminopeptidase, and α-glucosidase and negative for α-chymotrypsin, α-galactosidase, β-galactosidase, β-glucuronidase, β-glucosidase, *N*-acetyl-β-glucosaminidase, α-mannosidase, α-fucosidase, esterase lipase (C8), and trypsin. In the API 20NE tests, bacteria are positive for utilization of D-glucose, L-arabinose, D-mannitol, D-maltose, potassium gluconate, adipic acid, malic acid, trisodium citrate, and phenylacetic acid and negative for arginine dihydrolase, β-glucosidase, gelatinase, lysine decarboxylase, ornithine decarboxylase, urease, tryptophan deaminase, and utilization of D-mannose, *N*-acetyl-glucosamine, and capric acid. The predominant respiratory quinone is Q-9. The principal cellular fatty acids are summed feature 8 (C_18:1_ *ω*7*c*), C_16:0_, and C_19:0_ cyclo *ω*8*c*. The polar lipids are DPG, PG, PE, PL, and six unknown polar lipids. The genomic G+C content of the type strain is 63.9%.

The type strain BC-M4-5^T^ ( = MCCC 1A14433^T^ = KCTC 72092^T^) was isolated from sulfide samples in Tianxiu hydrothermal vents, on the Northwest Indian Ridge, Indian Ocean.

## Conclusion

In the present study, we isolated and established six novel species of *Halomonas* and classified them into the “*H. desiderata* group.” All six species as well as other members of this group were featured with aerobic denitrification and heterotrophic sulfide oxidation, attributed to the complete denitrification pathways and the occurrence of the *sqr/fccAB* genes, which encode sulfide-oxidizing enzymes and were widely distributed in the whole genus. In addition, the widespread occurrence of *tsdA* genes throughout the genus *Halomonas* was consistent with their ability of thiosulfate oxidation. This highlights the popularity of aerobic denitrification and heterotrophic sulfur oxidation among *Halomonas* bacteria, especially the group of “*H. desiderata*.” These insights help to explain their wide distribution in different environments and highlight their application potential for the removal of nitrogen and sulfide.

## Materials and Methods

### Strains and Culture Conditions

Thirteen *Halomonas* strains from marine environments and one from a municipal wastewater treatment plant were isolated in this study and deposited in the Marine Culture Collection of China (MCCC). As listed in Table 1, five of them are from deep sea sediments of the South China Sea and the Pacific Ocean, four from hydrothermal vent sulfide of the Northwest Indian Ocean, three from coastal water and sediments of the Taiwan Strait, two from sediments of shrimp culture ponds (Zhangzhou, China), and one from sewage of a municipal wastewater treatment plant (Xiamen, China). Unless otherwise specified, strains were grown aerobically on MA 2216 and in marine broth 2216 (BD, Difco) at 32°C. In addition, six type strains were also included. *Halomonas desiderata* DSM 9502^T^, *Halomonas campisalis* DSM 15413^T^, *Halomonas kenyensis* DSM 17331^T^, and *Halomonas elongata* DSM 2581^T^ were purchased from the Deutsche Sammlung von Mikroorganismen und Zellkulturen (DSMZ). *Halomonas daqingensis* CGMCC 1.6443^T^ was obtained from the China General Microbiological Culture Collection Center (CGMCC). *Halomonas saliphila* LCB169^T^ and *Halomonas lactosivorans* KCTC 52281^T^ were denoted by Yong-qiang Tian and Guo-xing Nie, respectively. These type strains were used for comparison of phenotypic, physiological, and chemotaxonomic characteristics and genomic analysis with the isolated strains in this study.

### Whole-Genome Sequence Analysis

Genomic DNA was extracted from each strain using the SBS extraction kit (SBS Genetech Co., Ltd., Shanghai, China) according to the manufacturer’s instructions. Purified genomic DNA was quantified by a NanoDrop 2000 spectrophotometer (Thermo Scientific). For strain MCCC 1A14433^T^, the genome was sequenced using a combination of PacBio RSII and Illumina sequencing platforms by Shanghai Majorbio Bio-pharm Technology Co., Ltd. (Shanghai, China). The assembly was produced using Canu software (Koren et al., 2017). The final assembly generated a circular genome with no gap. For strains MCCC 1A13718^T^ and MCCC 1A11059^T^, their genome sequences were determined by Tianjin Biochip Corporation using the PacBio sequencing platform. Sequencing reads were assembled using the hierarchical genome assembly process (HGAP 4.0) to obtain circular genomes. Four type strains (*H. campisalis* DSM 15413^T^, *H. kenyensis* DSM 17331^T^, *Halomonas neptunia* DSM15720^T^, and *Halomonas utahensis* DSM3051^T^), which lack available genomic information, as well as the other 11 isolated strains (as listed in Table 1), were sequenced on the Illumina HiSeq platform by Shanghai Majorbio Bio-pharm Technology Co., Ltd. (Shanghai, China). The high-quality reads were assembled by the software SPAdes version 3.12.0 with default parameters (Bankevich et al., 2012). All of the assembled genomes of these strains were deposited in the GenBank database, with the accession numbers as listed in Supplementary Table 1. Assembly features, including genome size, the number of contigs, and the G+C content, are summarized in Supplementary Table 1. The tRNA and rRNAs genes were searched using tRNAscan-SE software (v 2.0) (Lowe and Eddy, 1997) and RNAmmer (v 1.2) (Lagesen et al., 2007), respectively.

### Genome Collection and Analyses

For the phylogenomic tree construction and comparative genomic analysis, 253 publicly available *Halomonas* genomes in the GenBank database were collected, 76 of which were type strains. To obtain high-quality genomes, the genomes which were used for further analysis in this study met the criteria of an estimated completeness of >90% (except for type strains of *Halomonas sulfidaeris* ATCC BAA-803^T^ and *Halomonas olivaria* TYRC17^T^), an estimated contamination of <5%, and predicted contigs <200. Quality assessment of these genomes was conducted using the bioinformatic tools of CheckM (v1.0.12) and Quast (v5.0.0) (Gurevich et al., 2013) prior to annotation using Prokka (v1.12) (Seemann, 2014) and the RAST server (Aziz et al., 2008). After the evaluation of quality, a total of 207 genomic sequences were chosen for further comparative analysis. EggNOG-mapper was further used for functional annotation of the 207 genomes (Huerta-Cepas et al., 2017). The complete list of genomes used in this study is provided in Supplementary Table 1.

### Phylogenetic Analysis Based on 16S rRNA, *gyrB*, and *rpoD* Genes and Genomic Sequences

The 16S rRNA, *gyrB*, and *rpoD* gene sequences of the 14 strains were derived from the genomes. Other 16S rRNA, *gyrB*, and rpoD gene sequences of type strains within the genus *Halomonas* were obtained from the EzBiocloud database and National Center for Biotechnology Information (NCBI). Phylogenetic trees based on the concatenation of 16S rRNA, *gyrB*, and *rpoD* gene sequences were constructed using MEGA version 7 after multiple alignments of the data using the software MUSCLE v3.8.31 (Katoh and Standley, 2013), with distance options according to the Kimura two-parameter model and clustering with the neighbor-joining (NJ) method (Kumar et al., 2016). Bootstrap analysis based on 1,000 replications was used to estimate the confidence level of the tree topologies. *Carnimonas nigrificans* CECT 4437^T^ was used as outgroup in the phylogenetic analyses.

To determine the phylogenomic positions of the 14 strains, genome-based phylogenetic trees were reconstructed using two methods including PhyloPhlAn 3.0 (Nicola et al., 2013) and an up-to-date bacterial core gene set (UBCG) consisting of 92 genes (Na et al., 2018) using default settings, respectively. The visualization, annotation, and management of the phylogenomic trees were performed using the web-based tool evolview v3 (Subramanian et al., 2019)^2^.

### Overall Genome Relatedness Index Calculations

The similarity between genomes was assessed using the overall genome relatedness index (OGRI), which is useful for species delineation. Thus, dDDH was estimated *in silico* using the Genome-to-Genome Distance Calculator (GGDC 2.0) with the BLAST method and recommended formula 2^3^ (Meier-Kolthoff et al., 2013). ANIs were calculated by BLAST (ANIb) in JSpeciesWS (Richter et al., 2016), fastANI v1.3 (Jain et al., 2018)^4^, and the standalone Orthologous Average Nucleotide Identity Tool (OAT) (Lee et al., 2016). The visualization of the numerical matrix for dDDH and ANI values was carried out using TBtools v0.674 (Chen et al., 2020).

### Identification of Genes Involved in Nitrogen and Sulfur Metabolism

To compare the nitrogen metabolism pathways within the genus *Halomonas*, all 19 of the genomes sequenced in this study and 188 other genomes retrieved from NCBI were analyzed together. In these genomes, genes encoding proteins involved in denitrification (*NarGHIJ*, *NapABCDEFGH*, *NirSCDEFGHJLN*, *NirK*, *NorBCDE*, *qNor*, and *NosZYRLFD*), nitrate and nitrite assimilation (*NasA* and *NirBD*), and nitrate and nitrite transporters (*NrtABC* and *NarK*) and regulators (*NarXL*, *Fnr*, *NasR*, and *NasST*) were identified by a local BLASTP protein similarity search, with the protein sequences listed in Supplementary Table 6 used as queries. For the BLASTP analysis, we used an amino acid similarity cutoff of –40%, alignment coverage >80%, and an e-value cutoff of 1E-5. The genes encoding sulfur oxidation proteins, including *SoxXZYABCD* (Friedrich et al., 2001), *Sqr* (SQR) (Marcia et al., 2009), *FccAB* (FCSD) (Chen et al., 1994), PDO (persulfide dioxygenase) (Liu et al., 2014), and *TsdA* (thiosulfate dehydrogenase) (Denkmann et al., 2012; Brito et al., 2015) were also analyzed in these genomes. The presence/absence of these genes in the genomes was further checked based on the functional annotation by RAST and EggNOG-mapper.

Putative homologous proteins of NarG, NapA, NirS, NirK, NorB, qNor, NosZ, Sqr, FccA, PDO, and TsdA were retrieved from each query genome (Supplementary Table 7) and then used to construct the respective phylogenetic tree. Reference sequences were obtained from NCBI. Phylogenetic trees based on NarG, NapA, NirS, NorB, NosZ, Sqr, FccA, PDO, and TsdA amino acid sequences were constructed using MEGA version 7 after multiple alignments using the software MUSCLE v3.8.31 (Katoh and Standley, 2013), with distance options according to the Kimura two-parameter model and clustering with the NJ method (Kumar et al., 2016). Bootstrap analysis based on 1,000 replications was used to estimate the confidence level of the tree topologies.

Besides, the obtained putative homologues from the genome were also blasted against the NCBI database to obtain information on the domains present, which was used as an extra confirmation of their functional gene identity. Then, the presence/absence of all these functional genes tested in all of the genomes was used for co-occurrence analysis (Supplementary Table 5).

### Phenotypic and Physiological Characterization

Cellular and colonial morphology, motility, Gram staining, anaerobic growth of representative strains, and catalase and oxidase activities were examined according to previously described methods (Liu et al., 2017). The ranges and optima of temperature, pH, and NaCl concentration were tested according to previously described methods (Lai et al., 2014). Hydrolysis of protein, starch, casein, DNA, Tweens (20, 40, 60, and 80), and cellulose was conducted using traditional methods described by Mata et al. (2002). Other biochemical tests were carried out using API 20NE, API ZYM, and API 20E strips (bioMérieux) according to the manufacturer’s instructions, with the modification of adjusting the NaCl concentration to 3.0% in all of the tests.

### Fatty Acid, Polar Lipid, and Quinone Characterization

For whole-cell fatty acid analysis, cells were harvested from the third quadrants on MA medium at 32°C. The cells were saponified, methylated, and extracted using the standard MIDI (Sherlock Microbial Identification System, version 6.0B) protocol. The fatty acids were then analyzed by gas chromatography (GC) (Agilent Technologies 6850) and identified using the TSBA6.0 database of the Microbial Identification System. Polar lipids were extracted using a chloroform/methanol system and analyzed by one- and two-dimensional thin-layer chromatography (TLC), as described previously (Kates, 1986). Merck silica gel 60 F_254_ aluminum-backed thin-layer plates were used in TLC analyses. The plate dotted with sample was subjected to two-dimensional development, with the first solvent being chloroform–methanol–water (65:25:4, by vol.) and the second solvent being chloroform–methanol–acetic acid–water (85:12:15:4, by vol.). Total lipids were detected using molybdatophosphoric acid, and specific functional groups were detected using spray reagents specific for defined functional groups (Kates, 1986). Analysis of the respiratory quinones was carried out according to previously described methods (Collins, 1985).

### Assays of Denitrification Ability

Denitrification was determined in aerobic and anaerobic conditions, using a denitrification medium containing the following ingredients (per liter distilled water): sodium acetate 1.46 g, sodium succinate 2.40 g, sodium citrate 1.76 g, KH_2_PO_4_ 0.50 g, Na_2_HPO_4_ 0.50 g, MgSO_4_⋅7H_2_O 0.05 g, FeSO_4_⋅7H_2_O 0.002 g, CaCl_2_ 0.02 g, NaCl 30 g, NaNO_3_ 1.275 g (or NaNO_2_ 1.035 g), and trace element solution 1.0 ml, pH 7.5. The composition of trace element solution was described by Joo et al. (2005). The denitrification ability in anaerobic condition was examined in 100-ml sealed serum bottles containing 30 ml of medium, with the headspace filled with 100% He. The aerobic denitrification performance was examined in 500-ml sealed serum bottles containing 50 ml of medium, with the headspace filled with 75% He and 25% O_2_. The performance of aerobic denitrification of the six novel species was tested at 37°C with 15 mM ^15^N-labeled NaNO_3_ or NaNO_2_ as the sole nitrogen source and sodium citrate as the electron donor, at a C/N ratio of 8.0 and pH 7.5. Nitrate, nitrite, cell OD_600_, and gaseous nitrogen products (^15^N_2_ and N_2_O) as well as the oxygen percentage were analyzed during the experiment. All of the tests were performed in triplicate. ^30^N_2_ was analyzed by GC/isotope ratio mass spectrometry (GC/IRMS), and N_2_O and O_2_ were analyzed by GC/mass spectrometry (GC/MS) (Agilent 7890B). Besides, cell growth and the aerobic denitrification ability of the six novel species were tested with different electron donors, including acetate, sodium citrate, glucose, sucrose, lactate, glycerol, mannitol, maltose, ethanol, succinate, propionate, potassium sodium tartrate, sodium formate, pyruvate, methanol, formic acid, and acetic acid.

### Sulfur Oxidation Ability Test

Sulfide and thiosulfate oxidation tests were carried out according to previously described methods (Hou et al., 2018). Bacteria were cultivated to stationary phase, harvested by centrifugation (6,000 g, 5 min), and resuspended in 50 mM of HEPES buffer (pH 7.5) to a turbidity of 2 at 600 nm. Cell suspension (10 ml) was transferred to a 50-ml centrifuge tube. Freshly prepared sodium sulfide or thiosulfate was added to initiate the reaction. The tube was capped tightly and incubated at 30°C with shaking at 100 rpm. Sulfide and the products were analyzed at various time intervals. All of the tests were performed in triplicate. Sulfide was detected by a colorimetric method (Kamyshny, 2009). Thiosulfate, sulfate, tetrathionate, and sulfite were detected by ion chromatography (ICS-1100 system; Dionex).

Sulfide tolerance tests were also performed using denitrification medium containing NaNO_3_ as the sole nitrogen source and sodium citrate as the carbon source under aerobic conditions at 28°C, 150 rpm and pH 7.5. The sulfide concentration in the denitrification medium ranged from 1 to 15 mM. Nitrate, nitrite, sulfide, and cell growth were measured during the experiment. The sulfide oxidation ability was also tested under anaerobic conditions using denitrification medium, with sodium citrate as the sole electron donor and 15 mM of NaNO_3_ or 10% N_2_O (the headspace of the sealed vial was filled with 90% He and 10% N_2_O) as the sole electron acceptor.

## Data Availability Statement

The datasets presented in this study can be found in online repositories. The names of the repository/repositories and accession number(s) can be found in the article/Supplementary Material.

## Author Contributions

LW and ZS designed the experiment and reviewed the manuscript. LW performed the experiment, analyzed the data, and wrote the manuscript. Both authors contributed to the article and approved the submitted version.

## Conflict of Interest

The authors declare that the research was conducted in the absence of any commercial or financial relationships that could be construed as a potential conflict of interest.

## Footnotes

1 https://lpsn.dsmz.de/genus/halomonas

2 https://www.evolgenius.info/evolview/

3 http://ggdc.dsmz.de/distcalc2.php

4 https://github.com/ParBLiSS/FastANI/releases

## Supplementary Material

Supplementary Figure 1 Genome-based phylogenomic tree of the 14 strains along with 193 other *Halomonas* genomes and occurrence of genes for nitrogen, sulfur, and other metabolism. Phylogenomic tree was constructed using PhyloPhlAn 3.0. Strains shown in red are organisms isolated in this study. The visualization, annotation, and management of the phylogenomic trees were performed using the web-based tool evolview v3. Type strains of *Marinobacter hydrocarbonoclasticus* ATCC 49840^T^, *Alcanivorax dieselolei* B5^T^, and *Marinospirillum minutulum* DSM 6287^T^ were used as outgroup. The 31 right-most columns represent the distribution of key functional genes for nitrogen, sulfur, and other metabolism including carbon metabolism as well as stress responses. Blue represents the denitrification pathway; tan represents regulators for denitrification; green represents assimilation of nitrate and nitrite; fuchsia represents regulators for assimilation of nitrate and nitrite; orange represents the sulfur oxidation pathway; olive represents the PHA synthesis pathway; yellow represents phosphonate transporters and metabolism; dodgerblue represents the ectoine and glycine betaine synthesis pathway; gray represents arsenical oxidation-, reduction-, and resistance-related genes.

Supplementary Figure 2 Genome-based phylogenomic tree constructed based on the 14 strains along with 193 other *Halomonas* genomes using an up-to-date bacterial core gene (UBCG) set consisting of 92 genes. Strains shown in red are organisms isolated in this study. The visualization, annotation, and management of the phylogenomic trees were performed using the web-based tool evolview v3. Type strains of *Marinobacter hydrocarbonoclasticus* ATCC 49840^T^, *Alcanivorax dieselolei* B5^T^, and *Marinospirillum minutulum* DSM 6287^T^ were used as the outgroup.

Supplementary Figure 3 Transmission electron micrograph of negatively stained cells of six novel strains grown on MA 2216 (BD) for 12 h. **(A)** MCCC 1A11036^T^. **(B)** MCCC 1A11058^T^. **(C)** MCCC 1A11059^T^. **(D)** MCCC 1A11081^T^. **(E)** MCCC 1A13718^T^. **(F)** MCCC 1A14433^T^.

Supplementary Figure 4 Polar lipid profile of the six novel strains. **(A)** MCCC 1A11036^T^. **(B)** MCCC 1A11058^T^. **(C)** MCCC 1A11059^T^. **(D)** MCCC 1A11081^T^. **(E)** MCCC 1A13718^T^. **(F)** MCCC 1A14433^T^. DPG, diphosphatidylglycerol; PE, phosphatidylethanolamine; PG, phosphatidylglycerol; PL, unidentified phospholipid; PC, phosphatidylcholine; AL, unidentified aminolipid; APL, aminophospholipid; L, unknown polar lipid; F-first dimension of TLC; S, second dimension of TLC.

Supplementary Figure 5 Neighbor-joining phylogenetic tree based on the cytochrome cd1-type nitrite reductase NirS obtained from 64 *Halomonas* genomes and reference sequences. Bootstrap values (>50%) are shown at branch points. Bar, 0.1 nucleotide substitution rate (Knuc) units. The phylogeny was inferred using MEGA version 7 and 1000 bootstrap replicates. NirS sequence from *Arcobacter trophiarum* LMG 25534 was used as outgroup. The label in red font means the sequences from strains isolated in this study; blue font means sequences obtained from genomes of the genus *Halomonas*.

Supplementary Figure 6 Neighbor-joining phylogenetic tree based on the periplasmic nitrate reductase NapA obtained from 53 *Halomonas* genomes and reference sequences. Bootstrap values (>50%) are shown at branch points. Bar, 0.05 nucleotide substitution rate (Knuc) units. The phylogeny was inferred using MEGA version 7 and 1000 bootstrap replicates. The NapA sequence from *Magnetospira* sp. QH-2 was used as outgroup. The label in red font means the sequences from strains isolated in this study; blue font means sequences obtained from genomes of the genus *Halomonas*.

Supplementary Figure 7 Neighbor-joining phylogenetic tree based on the membrane-bound respiratory nitrate reductase NarG obtained from 157 *Halomonas* genomes and reference sequences. Bootstrap values (>50%) are shown at branch points. Bar, 0.1 nucleotide substitution rate (Knuc) units. The phylogeny was inferred using MEGA version 7 and 1000 bootstrap replicates. The NarG sequence from *Haloarcula hispanica* ATCC 33960 was used as outgroup. The label in red font means the sequences from strains isolated in this study; blue font means sequences obtained from genomes of the genus *Halomonas*.

Supplementary Figure 8 Neighbor-joining phylogenetic tree based on the cytochrome c nitric oxide reductase c-Nor obtained from 87 *Halomonas* genomes and reference sequences. Bootstrap values (>50%) are shown at branch points. Bar, 0.1 nucleotide substitution rate (Knuc) units. The phylogeny was inferred using MEGA version 7 and 1000 bootstrap replicates. The quinol-dependent nitric oxide reductase sequence from *Marinobacter hydrocarbonoclasticus* ATCC 49840^T^ was used as outgroup. The label in red font means the sequences from strains isolated in this study; blue font means sequences obtained from genomes of the genus *Halomonas*.

Supplementary Figure 9 Neighbor-joining phylogenetic tree based on the nitrous oxide reductase NosZ obtained from 87 *Halomonas* genomes and reference sequences. Bootstrap values (>50%) are shown at branch points. Bar, 0.05 nucleotide substitution rate (Knuc) units. The phylogeny was inferred using MEGA version 7 and 1000 bootstrap replicates. The NosZ sequence from *Azoarcus* sp. BH72 was used as outgroup. The label in red font means the sequences from strains isolated in this study; blue font means sequences obtained from genomes of the genus *Halomonas*.

Supplementary Figure 10 Phylogenetic tree based on 16S rRNA gene sequences from the 14 strains and type strains within the genus *Halomonas* as well as 193 other *Halomonas* genomes. The type strain *Marinobacter hydrocarbonoclasticus* ATCC 49840^T^ was used as outgroup. Bootstrap values (>50%) are shown at branch points. Bar, 0.01 nucleotide substitution rate (Knuc) units. The phylogeny was inferred using MEGA version 7 and 1000 bootstrap replicates. Each type strain is marked by a superscript capital T.

Supplementary Figure 11 Neighbor-joining phylogenetic tree based on the flavocytochrome c sulfide dehydrogenase FccA obtained from 38 *Halomonas* genomes and reference sequences. Bootstrap values (>50%) are shown at branch points. Bar, 20 nucleotide substitution rate (Knuc) units. The phylogeny was inferred using MEGA version 7 and 1000 bootstrap replicates. The FccA sequence from *Paracoccus denitrificans* was used as outgroup. The label in red font means the sequences from strains isolated in this study; blue font means sequences obtained from genomes of the genus *Halomonas*.

Supplementary Figure 12 Neighbor-joining phylogenetic tree based on the sulfide:quinone oxidoreductase SQR obtained from 67 *Halomonas* genomes and reference sequences. Bootstrap values (>50%) are shown at branch points. Bar, 10 nucleotide substitution rate (Knuc) units. The phylogeny was inferred using MEGA version 7 and 1000 bootstrap replicates. The FccA sequence from *Paracoccus denitrificans* was used as outgroup. The label in red font means the sequences from strains isolated in this study; blue font means sequences obtained from genomes of the genus *Halomonas*; bold font represents biochemically characterized SQR sequences.

Supplementary Figure 13 Partial sequence alignment of SQR homologous proteins from the genus *Halomonas* and reference species. The *Aquifex aeolicus* SQR sequence was used as a template for the conserved residues analysis. Strictly conserved residues are marked with asterisks. Reference sequences: sqrIV.Aquifex_aeolicus (UniParc ID: UPI00000567FF), sqrII-Pseudomonas_putida (UniParc ID: UPI000000E9AA), sqrI.Acidithiobacillus_ferrooxidans (UniParc ID: UPI0000562E8C), sqrI.Rhodobacter_capsulatus (UniParc ID: UPI0001D081BD), sqrIII.Caldivirga_maquilingensis (UniParc ID: UPI0000F24E05).

Supplementary Figure 14 Neighbor-joining phylogenetic tree based on the thiosulfate dehydrogenase TsdA obtained from 105 *Halomonas* genomes and reference sequences. Bootstrap values (>50%) are shown at branch points. Bar, 10 nucleotide substitution rate (Knuc) units. The phylogeny was inferred using MEGA version 7 and 1000 bootstrap replicates. The sulfur oxidation c-type cytochrome SoxA from *Paracoccus denitrificans* was used as outgroup. The label in red font means the sequences from strains isolated in this study; blue font means sequences obtained from genomes of the genus *Halomonas*; bold font represents functionally verified TsdA.

Supplementary Figure 15 Partial sequence alignment of TsdA homologous proteins from the genus *Halomonas* and reference species. Heme-binding motifs are indicated by red boxes, putative distal heme ligands are marked with red arrows. Strictly conserved residues are marked with asterisks. The TsdA sequence from *Allochromatium vinosum* DSM 180^T^ (ADC61061) was used as a template for the conserved domain and residues analysis.

Supplementary Figure 16 Neighbor-joining phylogenetic tree based on the persulfide dioxygenase PDO obtained from 203 *Halomonas* genomes and reference sequences. Bootstrap values (>50%) are shown at branch points. Bar, 5 nucleotide substitution rate (Knuc) units. The phylogeny was inferred using MEGA version 7 and 1000 bootstrap replicates. The glyoxalase sequence from *Haemophilus influenzae* R2846 was used as outgroup. The label in red font means the sequences from strains isolated in this study; blue font means sequences obtained from genomes of the genus *Halomonas*.
