# Supplementary material for: Aerobic Denitrification and Heterotrophic Sulfur Oxidation in the Genus Halomonas Revealed by Six Novel Species Characterizations and Genome-Based Analysis
Source: Front Microbiol. 2021 Mar 18;12:652766. doi: 10.3389/fmicb.2021.652766 (PMC8014003; doi:10.3389/fmicb.2021.652766)
Supplement: Supplementary Figure 1 — Genome-based phylogenomic tree of the 14 strains along with 193 other Halomonas genomes and occurrence of genes for nitrogen, sulfur, and other metabolism. Phylogenomic tree was constructed using PhyloPhlAn 3.0. Strains shown in red are organisms isolated in this study. The visualization, annotation, and management of the phylogenomic trees were performed using the web-based tool evolview v3. Type strains of Marinobacter hydrocarbonoclasticus ATCC 49840T, Alcanivorax dieselolei B5T, and Marinospirillum minutulum DSM 6287T were used as outgroup. The 31 right-most columns represent the distribution of key functional genes for nitrogen, sulfur, and other metabolism including carbon metabolism as well as stress responses. Blue represents the denitrification pathway; tan represents regulators for denitrification; green represents assimilation of nitrate and nitrite; fuchsia represents regulators for assimilation of nitrate and nitrite; orange represents the sulfur oxidation pathway; olive represents the PHA synthesis pathway; yellow represents phosphonate transporters and metabolism; dodgerblue represents the ectoine and glycine betaine synthesis pathway; gray represents arsenical oxidation-, reduction-, and resistance-related genes. [file Data_Sheet_1.zip › Figure S1-S16/Figure S10.pdf]

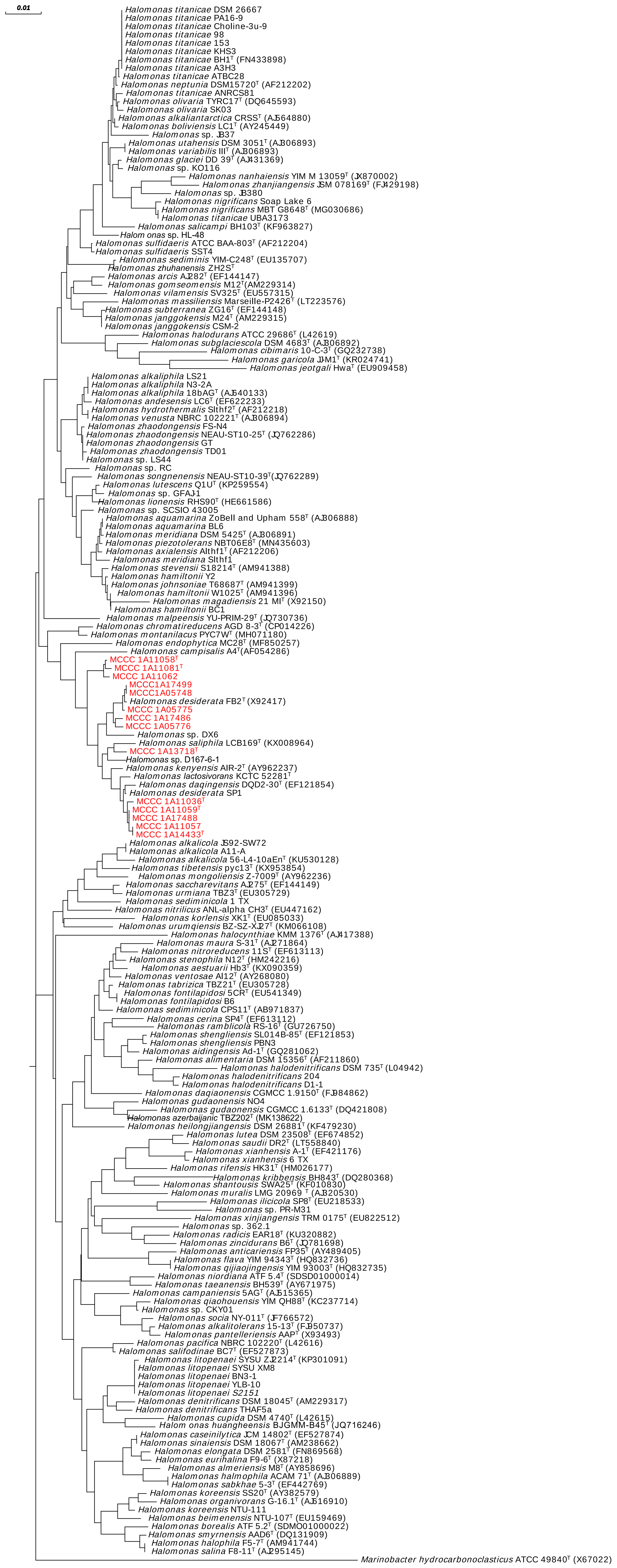
0.01
Halomonas titanicae DSM 26667
Halomonas titanicae PA16-9
Halomonas titanicae Choline-3u-9
Halomonas titanicae 98
Halomonas titanicae 153
Halomonas titanicae KHS3
Halomonas titanicae BH1^T (FN433898)
Halomonas titanicae A3H3
Halomonas titanicae ATBC28
Halomonas neptunia DSM15720^T (AF212202)
Halomonas titanicae ANRCS81
Halomonas olivaria TYRC17^T (DQ645593)
Halomonas olivaria SK03
Halomonas alkaliantarctica CRSS^T (AJ564880)
Halomonas boliviensis LC1^T (AY245449)
Halomonas sp. JB37
Halomonas utahensis DSM 3051^T (AJ306893)
Halomonas variabilis III^T (AJ306893)
Halomonas glaciei DD 39^T (AJ431369)
Halomonas sp. KO116
Halomonas nanhaiensis YIM M 13059^T (JX870002)
Halomonas zhanjiangensis JSM 078169^T (FJ429198)
Halomonas sp. JB380
Halomonas nigrificans Soap Lake 6
Halomonas nigrificans MBT G8648^T (MG030686)
Halomonas titanicae UBA3173
Halomonas salicampi BH103^T (KF963827)
Halomonas sp. HL-48
Halomonas sulfidaeris ATCC BAA-803^T (AF212204)
Halomonas sulfidaeris SST4
Halomonas sediminis YIM-C248^T (EU135707)
Halomonas zhuhanensis ZH2S^T
Halomonas arcis AJ282^T (EF144147)
Halomonas gomseomensis M12^T(AM229314)
Halomonas vilamensis SV325^T (EU557315)
Halomonas massiliensis Marseille-P2426^T (LT223576)
Halomonas subterranea ZG16^T (EF144148)
Halomonas janggokensis M24^T (AM229315)
Halomonas janggokensis CSM-2
Halomonas halodurans ATCC 29686^T (L42619)
Halomonas subglaciescola DSM 4683^T (AJ306892)
Halomonas cibimaris 10-C-3^T (GQ232738)
Halomonas garicola JJM1^T (KR024741)
Halomonas jeotgali Hwa^T (EU909458)
Halomonas alkaliphila LS21
Halomonas alkaliphila N3-2A
Halomonas alkaliphila 18bAG^T (AJ640133)
Halomonas andesensis LC6^T (EF622233)
Halomonas hydrothermalis Slthf2^T (AF212218)
Halomonas venusta NBRC 102221^T (AJ306894)
Halomonas zhaodongensis FS-N4
Halomonas zhaodongensis NEAU-ST10-25^T (JQ762286)
Halomonas zhaodongensis GT
Halomonas zhaodongensis TD01
Halomonas sp. LS44
Halomonas sp. RC
Halomonas songnenensis NEAU-ST10-39^T(JQ762289)
Halomonas lutescens Q1U^T (KP259554)
Halomonas sp. GFAJ-1
Halomonas lionensis RHS90^T (HE661586)
Halomonas sp. SCSIO 43005
Halomonas aquamarina ZoBell and Upham 558^T (AJ306888)
Halomonas aquamarina BL6
Halomonas meridiana DSM 5425^T (AJ306891)
Halomonas piezotolerans NBT06E8^T (MN435603)
Halomonas axialensis Althf1^T (AF212206)
Halomonas meridiana Slthf1
Halomonas stevensii S18214^T (AM941388)
Halomonas hamiltonii Y2
Halomonas johnsoniae T68687^T (AM941399)
Halomonas hamiltonii W1025^T (AM941396)
Halomonas magadiensis 21 MI^T (X92150)
Halomonas hamiltonii BC1
Halomonas malpeensis YU-PRIM-29^T (JQ730736)
Halomonas chromatireducens AGD 8-3^T (CP014226)
Halomonas montanilacus PYC7W^T (MH071180)
Halomonas endophytica MC28^T (MF850257)
Halomonas campisalis A4^T(AF054286)
MCCC 1A11058^T
MCCC 1A11081^T
MCCC 1A11062
MCCC1A17499
MCCC1A05748
Halomonas desiderata FB2^T (X92417)
MCCC 1A05775
MCCC 1A17486
MCCC 1A05776
Halomonas sp. DX6
Halomonas saliphila LCB169^T (KX008964)
MCCC 1A13718^T
Halomonas sp. D167-6-1
Halomonas kenyensis AIR-2^T (AY962237)
Halomonas lactosivorans KCTC 52281^T
Halomonas daqingensis DQD2-30^T (EF121854)
Halomonas desiderata SP1
MCCC 1A11036^T
MCCC 1A11059^T
MCCC 1A17488
MCCC 1A11057
MCCC 1A14433^T
Halomonas alkalicola JS92-SW72
Halomonas alkalicola A11-A
Halomonas alkalicola 56-L4-10aEn^T (KU530128)
Halomonas tibetensis pyc13^T (KX953854)
Halomonas mongoliensis Z-7009^T (AY962236)
Halomonas saccharevitans AJ275^T (EF144149)
Halomonas urmiana TBZ3^T (EU305729)
Halomonas sediminicola 1 TX
Halomonas nitrilicus ANL-alpha CH3^T (EU447162)
Halomonas korlensis XK1^T (EU085033)
Halomonas urumqiensis BZ-SZ-XJ27^T (KM066108)
Halomonas halocynthiae KMM 1376^T (AJ417388)
Halomonas maura S-31^T (AJ271864)
Halomonas nitroreducens 11S^T (EF613113)
Halomonas stenophila N12^T (HM242216)
Halomonas aestuarii Hb3^T (KX090359)
Halomonas ventosae Al12^T (AY268080)
Halomonas tabrizica TBZ21^T (EU305728)
Halomonas fontilapidosi 5CR^T (EU541349)
Halomonas fontilapidosi B6
Halomonas sediminicola CPS11^T (AB971837)
Halomonas cerina SP4^T (EF613112)
Halomonas ramblicola RS-16^T (GU726750)
Halomonas shengliensis SL014B-85^T (EF121853)
Halomonas shengliensis PBN3
Halomonas aidingensis Ad-1^T (GQ281062)
Halomonas alimentaria DSM 15356^T (AF211860)
Halomonas halodenitrificans DSM 735^T (L04942)
Halomonas halodenitrificans 204
Halomonas halodenitrificans D1-1
Halomonas daqiaonensis CGMCC 1.9150^T (FJ984862)
Halomonas gudaonensis NO4
Halomonas gudaonensis CGMCC 1.6133^T (DQ421808)
Halomonas azerbaijanic TBZ202^T (MK138622)
Halomonas heilongjiangensis DSM 26881^T (KF479230)
Halomonas lutea DSM 23508^T (EF674852)
Halomonas saudii DR2^T (LT558840)
Halomonas xianhensis A-1^T (EF421176)
Halomonas xianhensis 6 TX
Halomonas rifensis HK31^T (HM026177)
Halomonas kribbensis BH843^T (DQ280368)
Halomonas shantousis SWA25^T (KF010830)
Halomonas muralis LMG 20969 ^{T} (AJ320530)
Halomonas ilicicola SP8^T (EU218533)
Halomonas sp. PR-M31
Halomonas xinjiangensis TRM 0175^T (EU822512)
Halomonas sp. 362.1
Halomonas radicis EAR18^T (KU320882)
Halomonas zincidurans B6^T (JQ781698)
Halomonas anticariensis FP35^T (AY489405)
Halomonas flava YIM 94343^T (HQ832736)
Halomonas qijiaojingensis YIM 93003^T (HQ832735)
Halomonas niordiana ATF 5.4^T (SDSD01000014)
Halomonas taeanensis BH539^T (AY671975)
Halomonas campaniensis 5AG^T (AJ515365)
Halomonas qiaohouensis YIM QH88^T (KC237714)
Halomonas sp. CKY01
Halomonas socia NY-011^T (JF766572)
Halomonas alkalitolerans 15-13^T (FJ950737)
Halomonas pantelleriensis AAP^T (X93493)
Halomonas pacifica NBRC 102220^T (L42616)
Halomonas salifodinae BC7^T (EF527873)
Halomonas litopenaei SYSU ZJ2214^T (KP301091)
Halomonas litopenaei SYSU XM8
Halomonas litopenaei BN3-1
Halomonas litopenaei YLB-10
Halomonas litopenaei S2151
Halomonas denitrificans DSM 18045^T (AM229317)
Halomonas denitrificans THAF5a
Halomonas cupida DSM 4740^T (L42615)
Halom onas huangheensis BJGMM-B45^T (JQ716246)
Halomonas caseinilytica JCM 14802^T (EF527874)
Halomonas sinaiensis DSM 18067^T (AM238662)
Halomonas elongata DSM 2581^T (FN869568)
Halomonas eurihalina F9-6^T (X87218)
Halomonas almeriensis M8^T (AY858696)
Halomonas halmophila ACAM 71^T (AJ306889)
Halomonas sabkhae 5-3^T (EF442769)
Halomonas koreensis SS20^T (AY382579)
Halomonas organivorans G-16.1^T (AJ616910)
Halomonas koreensis NTU-111
Halomonas beimenensis NTU-107^T (EU159469)
Halomonas borealis ATF 5.2^T (SDMO01000022)
Halomonas smyrnensis AAD6^T (DQ131909)
Halomonas halophila F5-7^T (AM941744)
Halomonas salina F8-11^T (AJ295145)
Marinobacter hydrocarbonoclasticus ATCC 49840^T (X67022)
