# Supplementary material for: Aerobic Denitrification and Heterotrophic Sulfur Oxidation in the Genus Halomonas Revealed by Six Novel Species Characterizations and Genome-Based Analysis
Source: Front Microbiol. 2021 Mar 18;12:652766. doi: 10.3389/fmicb.2021.652766 (PMC8014003; doi:10.3389/fmicb.2021.652766)
Supplement: Supplementary Figure 1 — Genome-based phylogenomic tree of the 14 strains along with 193 other Halomonas genomes and occurrence of genes for nitrogen, sulfur, and other metabolism. Phylogenomic tree was constructed using PhyloPhlAn 3.0. Strains shown in red are organisms isolated in this study. The visualization, annotation, and management of the phylogenomic trees were performed using the web-based tool evolview v3. Type strains of Marinobacter hydrocarbonoclasticus ATCC 49840T, Alcanivorax dieselolei B5T, and Marinospirillum minutulum DSM 6287T were used as outgroup. The 31 right-most columns represent the distribution of key functional genes for nitrogen, sulfur, and other metabolism including carbon metabolism as well as stress responses. Blue represents the denitrification pathway; tan represents regulators for denitrification; green represents assimilation of nitrate and nitrite; fuchsia represents regulators for assimilation of nitrate and nitrite; orange represents the sulfur oxidation pathway; olive represents the PHA synthesis pathway; yellow represents phosphonate transporters and metabolism; dodgerblue represents the ectoine and glycine betaine synthesis pathway; gray represents arsenical oxidation-, reduction-, and resistance-related genes. [file Data_Sheet_1.zip › Figure S1-S16/Figure S13.pdf]

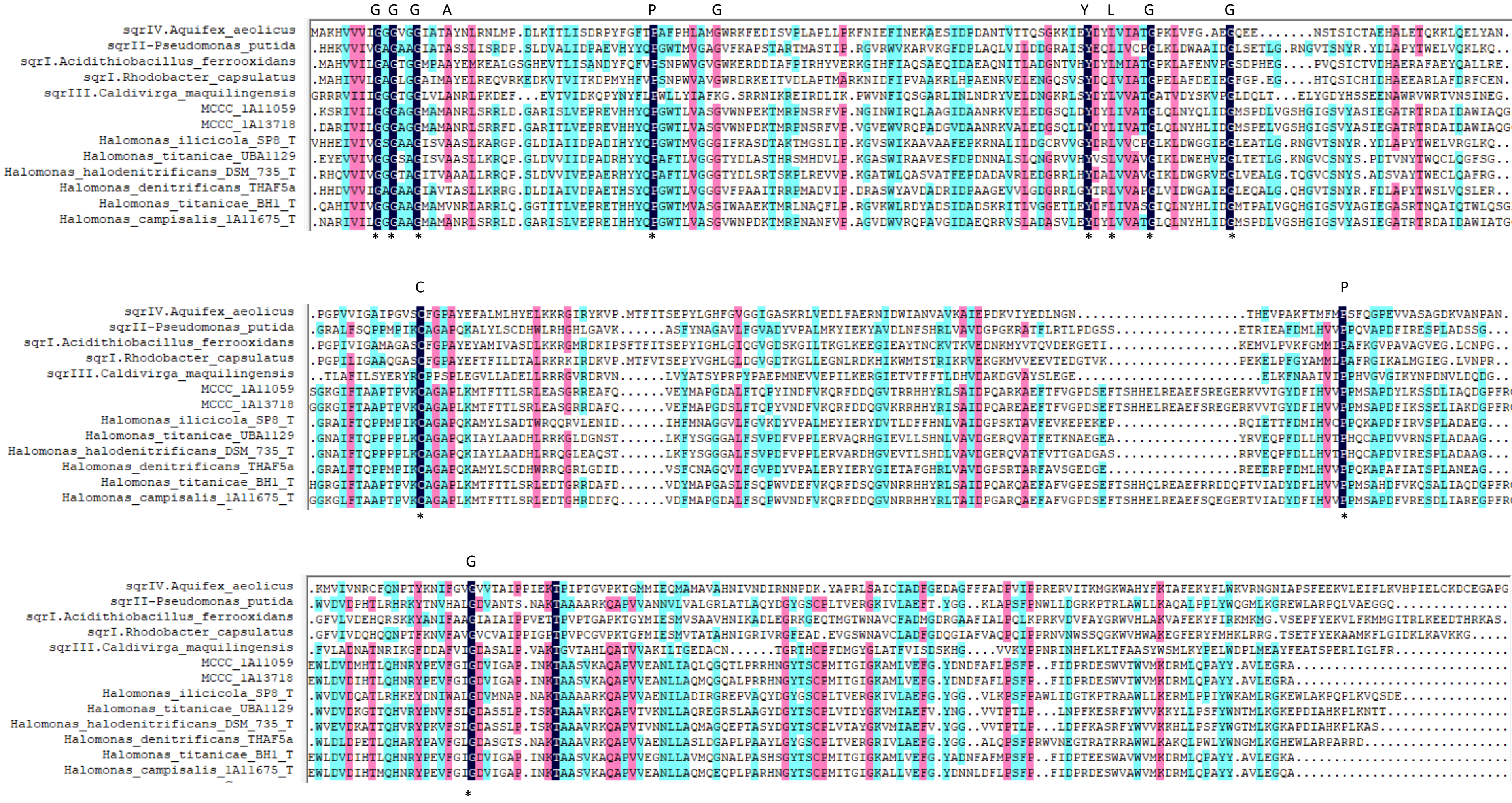
G G G A P G Y L G G
sqrIV.Aquifex_aeolicus
sqrII-Pseudomonas_putida
sqrI.Acidithiobacillus_ferrooxidans
sqrI.Rhodobacter_capsulatus
sqrIII.Caldivirga_maquilingensis
MCCC_1A11059
MCCC_1A13718
Halomonas_ilicicola_SP8_T
Halomonas_titanicae_UBA1129
Halomonas_halodenitrificans_DSM_735_T
Halomonas_denitrificans_THAF5a
Halomonas_titanicae_BH1_T
Halomonas_campisalis_1A11675_T
C P
sqrIV.Aquifex_aeolicus
sqrII-Pseudomonas_putida
sqrI.Acidithiobacillus_ferrooxidans
sqrI.Rhodobacter_capsulatus
sqrIII.Caldivirga_maquilingensis
MCCC_1A11059
MCCC_1A13718
Halomonas_ilicicola_SP8_T
Halomonas_titanicae_UBA1129
Halomonas_halodenitrificans_DSM_735_T
Halomonas_denitrificans_THAF5a
Halomonas_titanicae_BH1_T
Halomonas_campisalis_1A11675_T
G
sqrIV.Aquifex_aeolicus
sqrII-Pseudomonas_putida
sqrI.Acidithiobacillus_ferrooxidans
sqrI.Rhodobacter_capsulatus
sqrIII.Caldivirga_maquilingensis
MCCC_1A11059
MCCC_1A13718
Halomonas_ilicicola_SP8_T
Halomonas_titanicae_UBA1129
Halomonas_halodenitrificans_DSM_735_T
Halomonas_denitrificans_THAF5a
Halomonas_titanicae_BH1_T
Halomonas_campisalis_1A11675_T
