# Supplementary figures and images for: Aerobic Denitrification and Heterotrophic Sulfur Oxidation in the Genus Halomonas Revealed by Six Novel Species Characterizations and Genome-Based Analysis
Source: Front Microbiol. 2021 Mar 18;12:652766. doi: 10.3389/fmicb.2021.652766 (PMC8014003; doi:10.3389/fmicb.2021.652766)

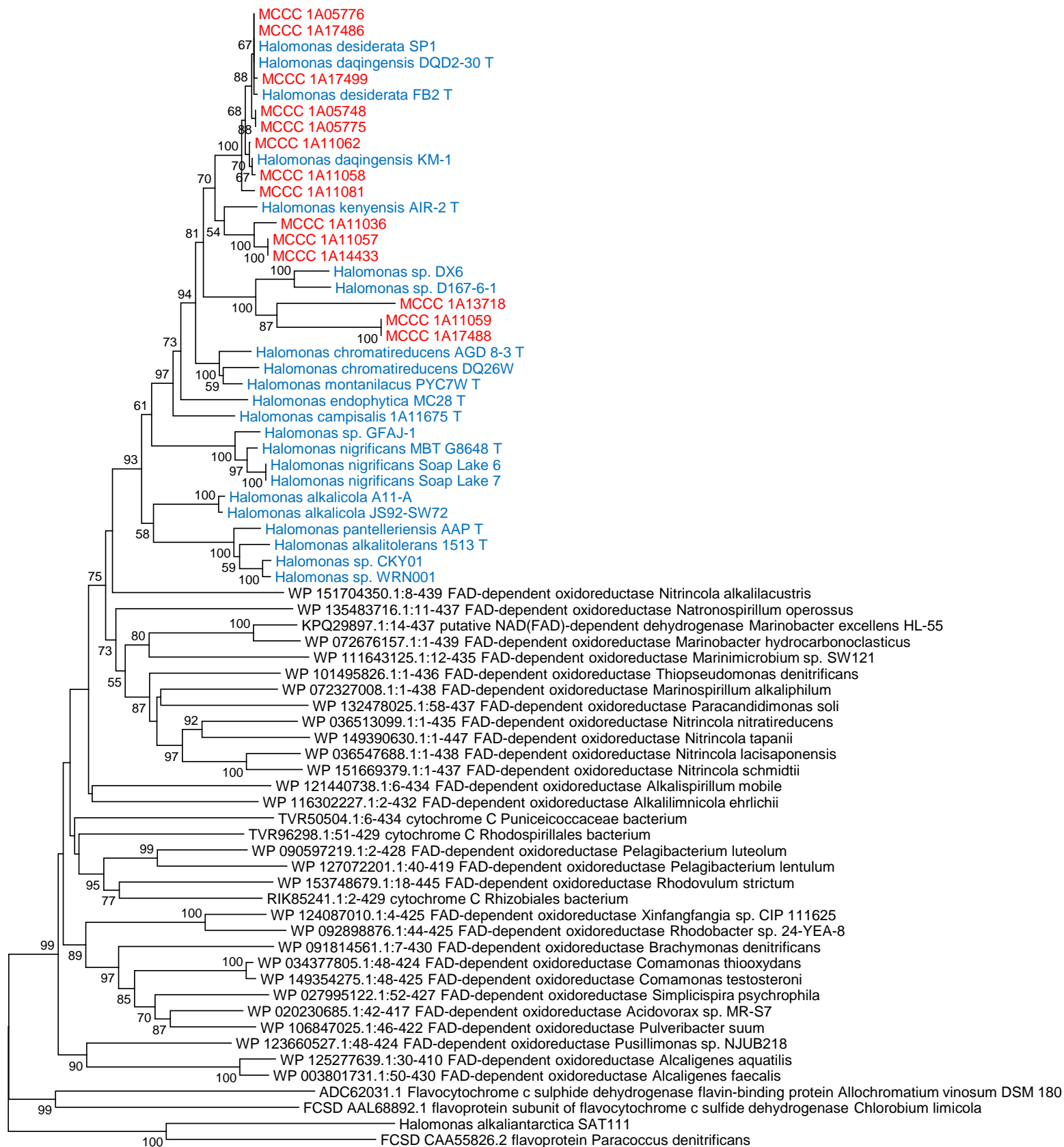

Supplement: Supplementary Figure 1 — Genome-based phylogenomic tree of the 14 strains along with 193 other Halomonas genomes and occurrence of genes for nitrogen, sulfur, and other metabolism. Phylogenomic tree was constructed using PhyloPhlAn 3.0. Strains shown in red are organisms isolated in this study. The visualization, annotation, and management of the phylogenomic trees were performed using the web-based tool evolview v3. Type strains of Marinobacter hydrocarbonoclasticus ATCC 49840T, Alcanivorax dieselolei B5T, and Marinospirillum minutulum DSM 6287T were used as outgroup. The 31 right-most columns represent the distribution of key functional genes for nitrogen, sulfur, and other metabolism including carbon metabolism as well as stress responses. Blue represents the denitrification pathway; tan represents regulators for denitrification; green represents assimilation of nitrate and nitrite; fuchsia represents regulators for assimilation of nitrate and nitrite; orange represents the sulfur oxidation pathway; olive represents the PHA synthesis pathway; yellow represents phosphonate transporters and metabolism; dodgerblue represents the ectoine and glycine betaine synthesis pathway; gray represents arsenical oxidation-, reduction-, and resistance-related genes. [file Data_Sheet_1.zip › Figure S1-S16/Figure S11.pdf]

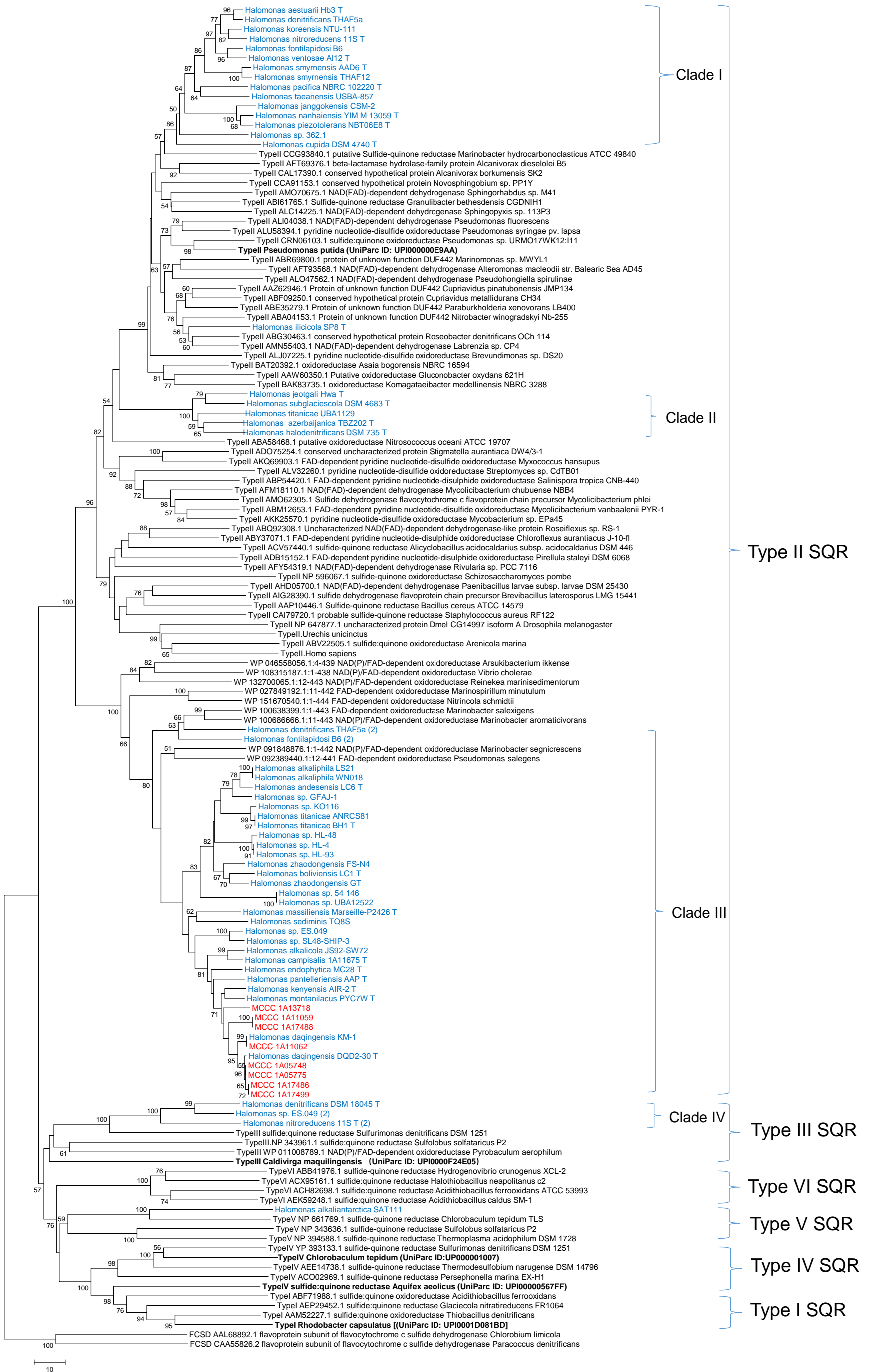

Supplement: Supplementary Figure 1 — Genome-based phylogenomic tree of the 14 strains along with 193 other Halomonas genomes and occurrence of genes for nitrogen, sulfur, and other metabolism. Phylogenomic tree was constructed using PhyloPhlAn 3.0. Strains shown in red are organisms isolated in this study. The visualization, annotation, and management of the phylogenomic trees were performed using the web-based tool evolview v3. Type strains of Marinobacter hydrocarbonoclasticus ATCC 49840T, Alcanivorax dieselolei B5T, and Marinospirillum minutulum DSM 6287T were used as outgroup. The 31 right-most columns represent the distribution of key functional genes for nitrogen, sulfur, and other metabolism including carbon metabolism as well as stress responses. Blue represents the denitrification pathway; tan represents regulators for denitrification; green represents assimilation of nitrate and nitrite; fuchsia represents regulators for assimilation of nitrate and nitrite; orange represents the sulfur oxidation pathway; olive represents the PHA synthesis pathway; yellow represents phosphonate transporters and metabolism; dodgerblue represents the ectoine and glycine betaine synthesis pathway; gray represents arsenical oxidation-, reduction-, and resistance-related genes. [file Data_Sheet_1.zip › Figure S1-S16/Figure S12.pdf]

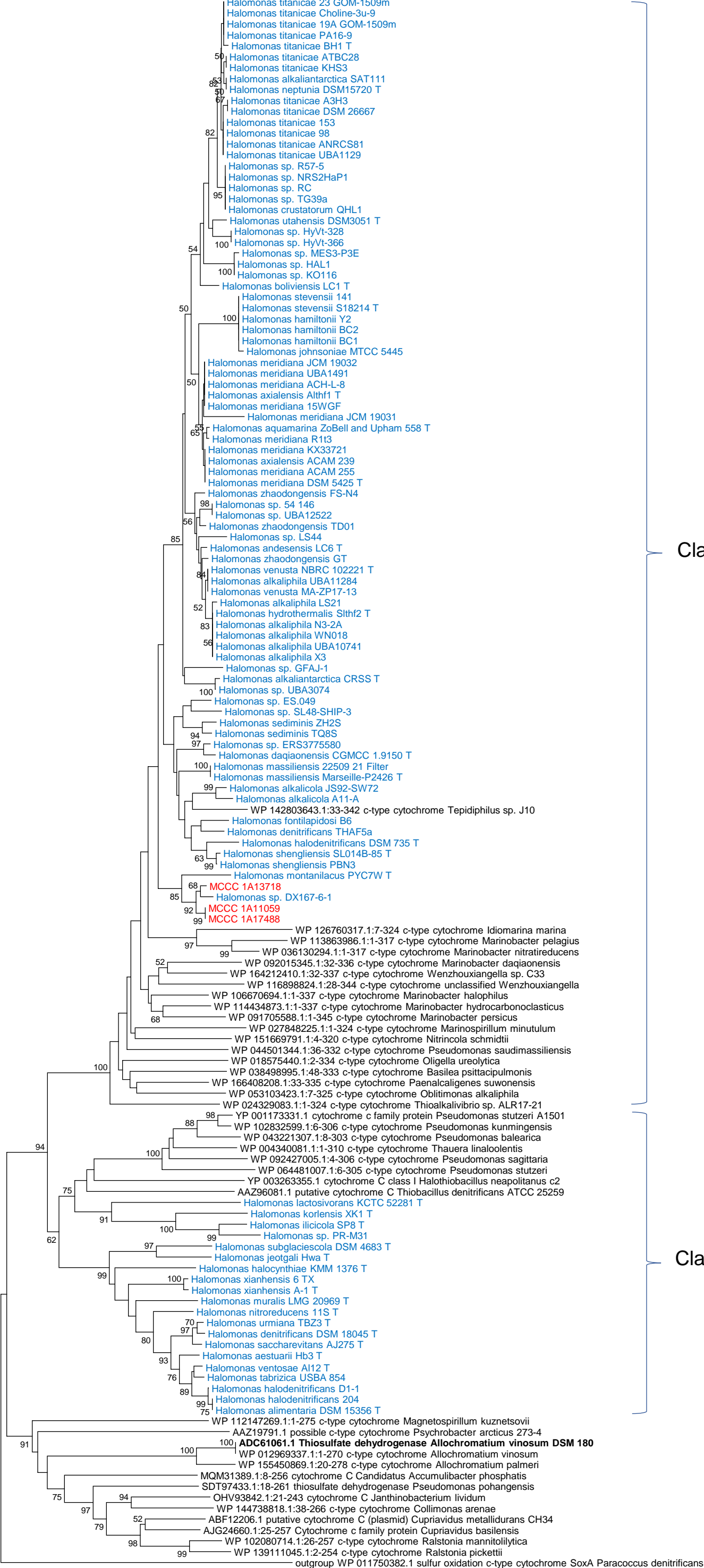

Clade I

Clade II

Supplement: Supplementary Figure 1 — Genome-based phylogenomic tree of the 14 strains along with 193 other Halomonas genomes and occurrence of genes for nitrogen, sulfur, and other metabolism. Phylogenomic tree was constructed using PhyloPhlAn 3.0. Strains shown in red are organisms isolated in this study. The visualization, annotation, and management of the phylogenomic trees were performed using the web-based tool evolview v3. Type strains of Marinobacter hydrocarbonoclasticus ATCC 49840T, Alcanivorax dieselolei B5T, and Marinospirillum minutulum DSM 6287T were used as outgroup. The 31 right-most columns represent the distribution of key functional genes for nitrogen, sulfur, and other metabolism including carbon metabolism as well as stress responses. Blue represents the denitrification pathway; tan represents regulators for denitrification; green represents assimilation of nitrate and nitrite; fuchsia represents regulators for assimilation of nitrate and nitrite; orange represents the sulfur oxidation pathway; olive represents the PHA synthesis pathway; yellow represents phosphonate transporters and metabolism; dodgerblue represents the ectoine and glycine betaine synthesis pathway; gray represents arsenical oxidation-, reduction-, and resistance-related genes. [file Data_Sheet_1.zip › Figure S1-S16/Figure S14.pdf]

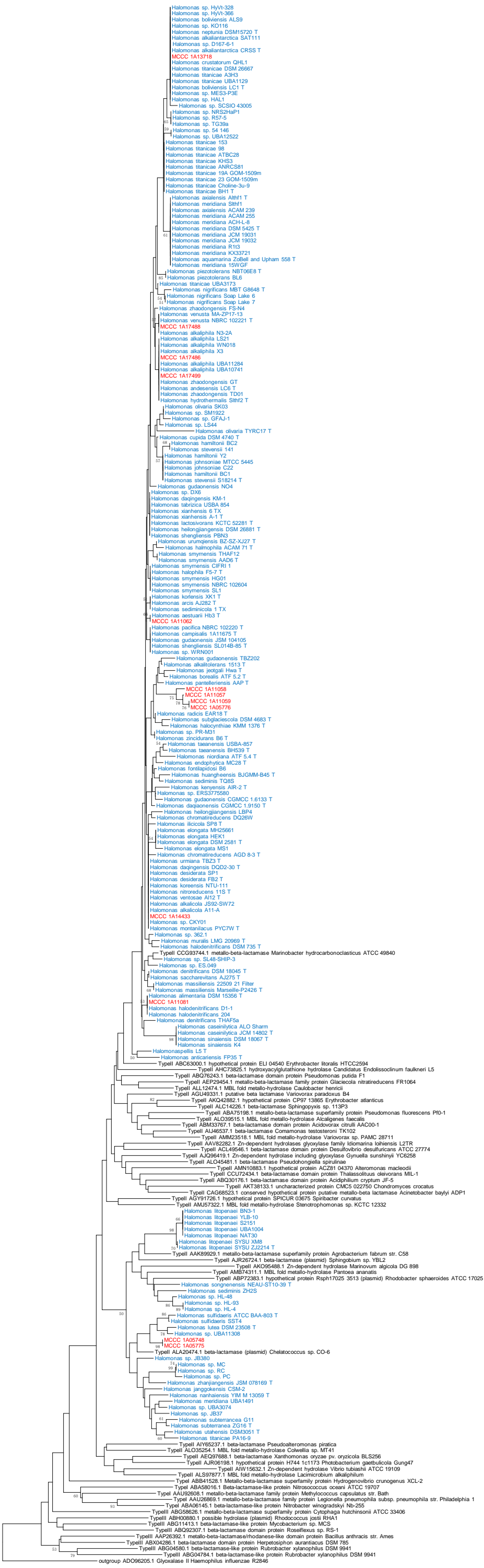

pdo I

Pdo II

Pdo III

Supplement: Supplementary Figure 1 — Genome-based phylogenomic tree of the 14 strains along with 193 other Halomonas genomes and occurrence of genes for nitrogen, sulfur, and other metabolism. Phylogenomic tree was constructed using PhyloPhlAn 3.0. Strains shown in red are organisms isolated in this study. The visualization, annotation, and management of the phylogenomic trees were performed using the web-based tool evolview v3. Type strains of Marinobacter hydrocarbonoclasticus ATCC 49840T, Alcanivorax dieselolei B5T, and Marinospirillum minutulum DSM 6287T were used as outgroup. The 31 right-most columns represent the distribution of key functional genes for nitrogen, sulfur, and other metabolism including carbon metabolism as well as stress responses. Blue represents the denitrification pathway; tan represents regulators for denitrification; green represents assimilation of nitrate and nitrite; fuchsia represents regulators for assimilation of nitrate and nitrite; orange represents the sulfur oxidation pathway; olive represents the PHA synthesis pathway; yellow represents phosphonate transporters and metabolism; dodgerblue represents the ectoine and glycine betaine synthesis pathway; gray represents arsenical oxidation-, reduction-, and resistance-related genes. [file Data_Sheet_1.zip › Figure S1-S16/Figure S16.pdf]

0.1

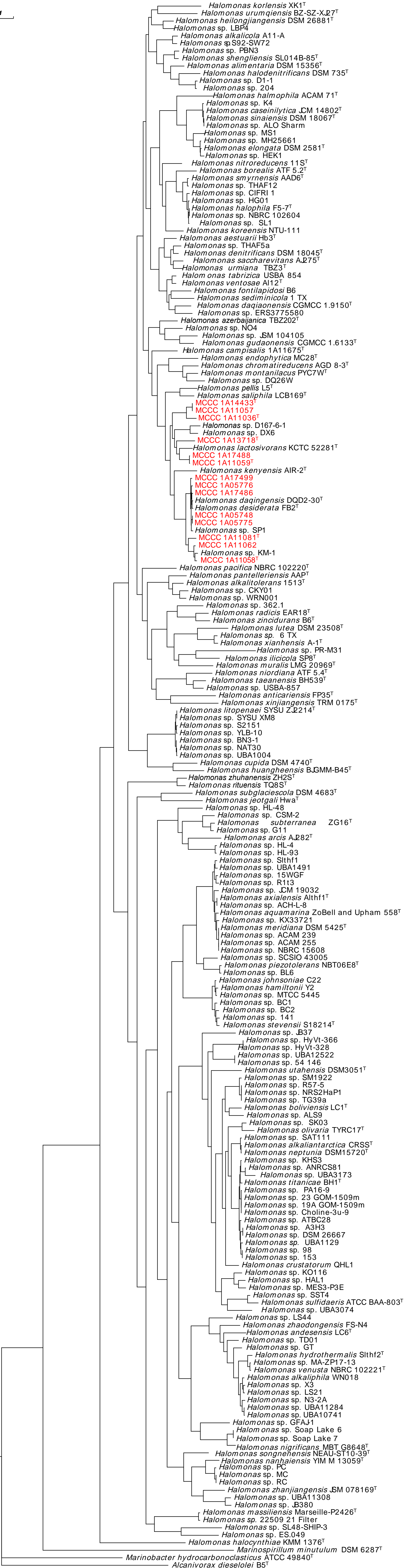

Supplement: Supplementary Figure 1 — Genome-based phylogenomic tree of the 14 strains along with 193 other Halomonas genomes and occurrence of genes for nitrogen, sulfur, and other metabolism. Phylogenomic tree was constructed using PhyloPhlAn 3.0. Strains shown in red are organisms isolated in this study. The visualization, annotation, and management of the phylogenomic trees were performed using the web-based tool evolview v3. Type strains of Marinobacter hydrocarbonoclasticus ATCC 49840T, Alcanivorax dieselolei B5T, and Marinospirillum minutulum DSM 6287T were used as outgroup. The 31 right-most columns represent the distribution of key functional genes for nitrogen, sulfur, and other metabolism including carbon metabolism as well as stress responses. Blue represents the denitrification pathway; tan represents regulators for denitrification; green represents assimilation of nitrate and nitrite; fuchsia represents regulators for assimilation of nitrate and nitrite; orange represents the sulfur oxidation pathway; olive represents the PHA synthesis pathway; yellow represents phosphonate transporters and metabolism; dodgerblue represents the ectoine and glycine betaine synthesis pathway; gray represents arsenical oxidation-, reduction-, and resistance-related genes. [file Data_Sheet_1.zip › Figure S1-S16/Figure S2.pdf]

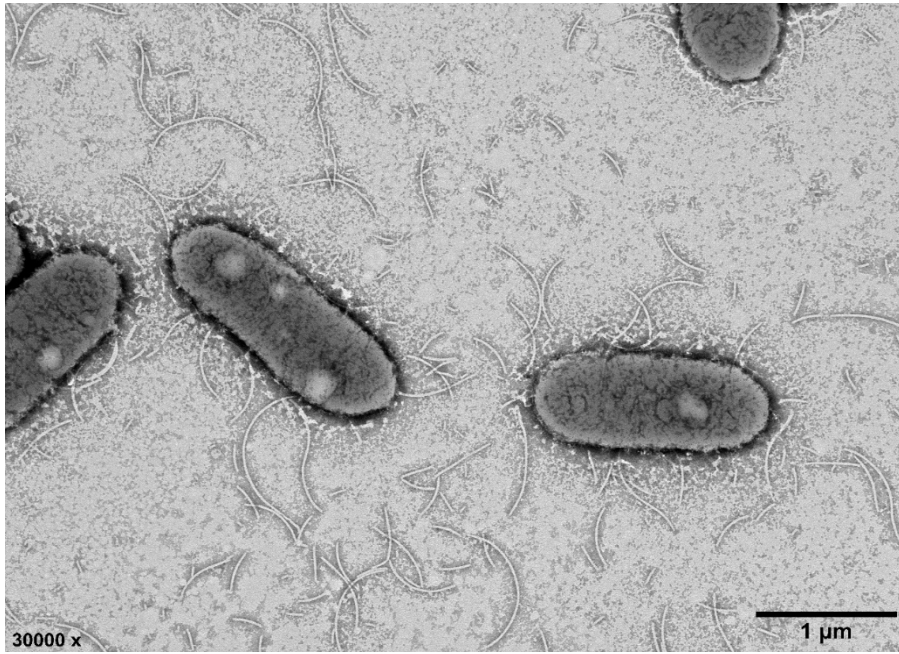

a

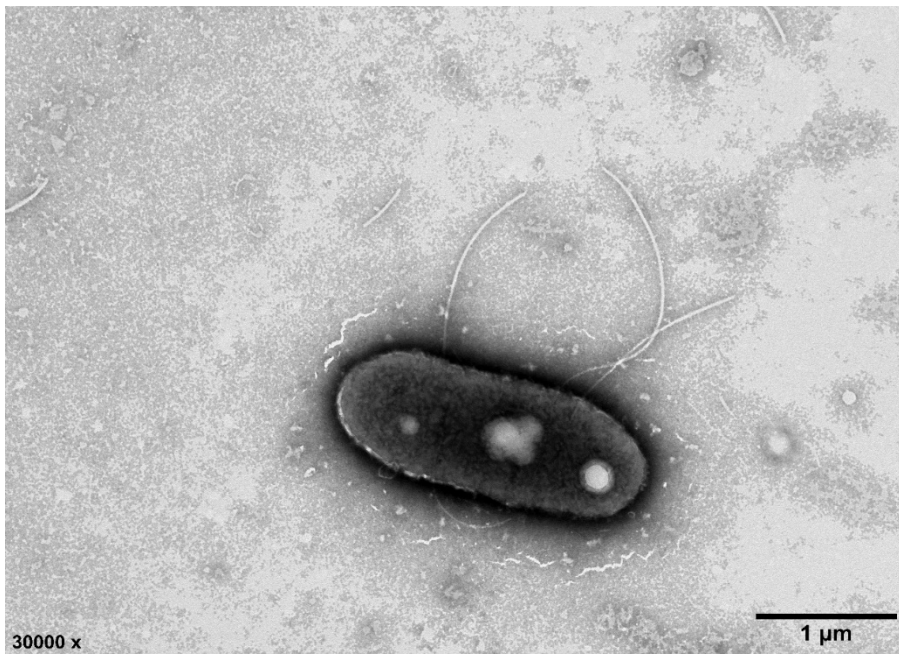

b

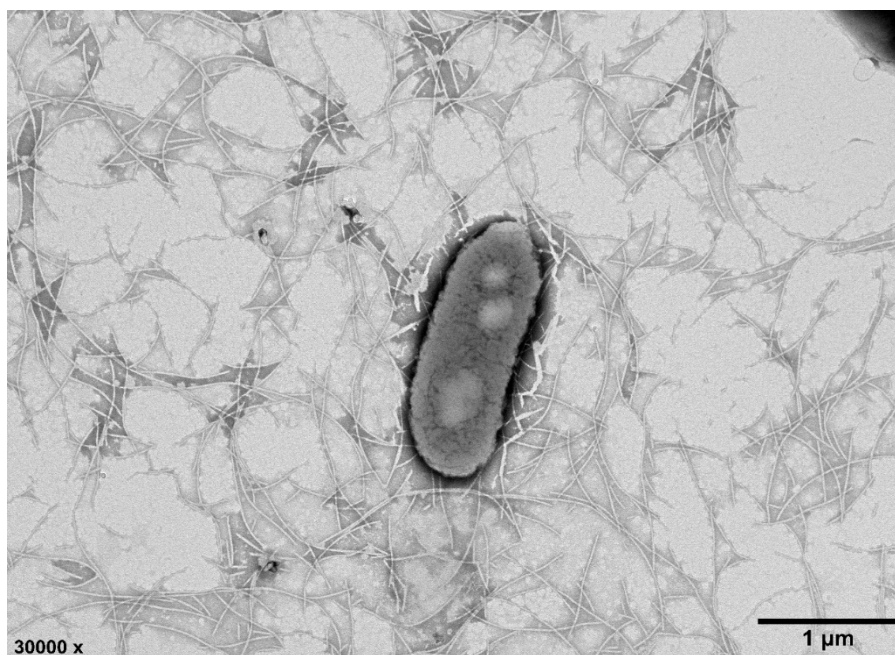

c

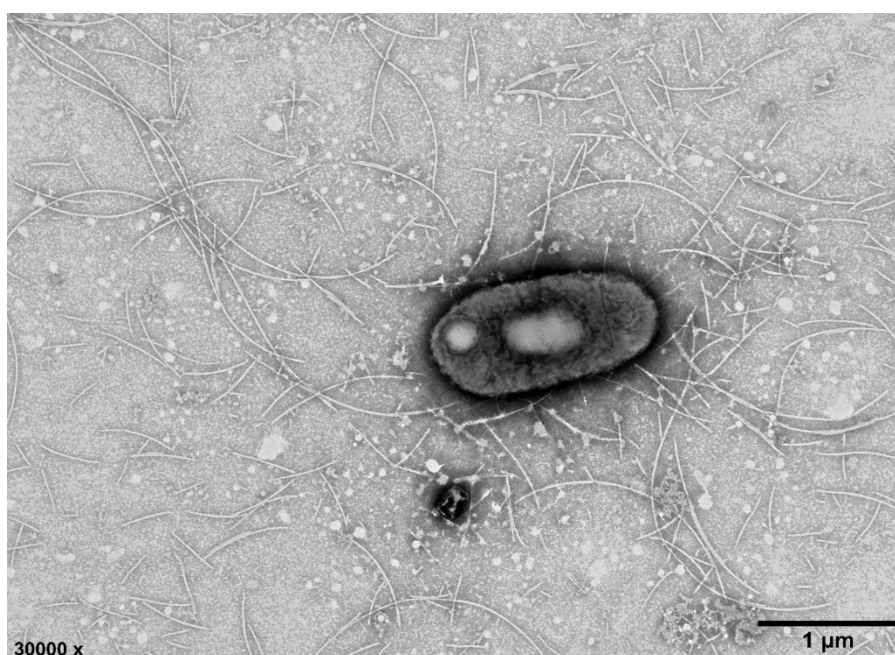

d

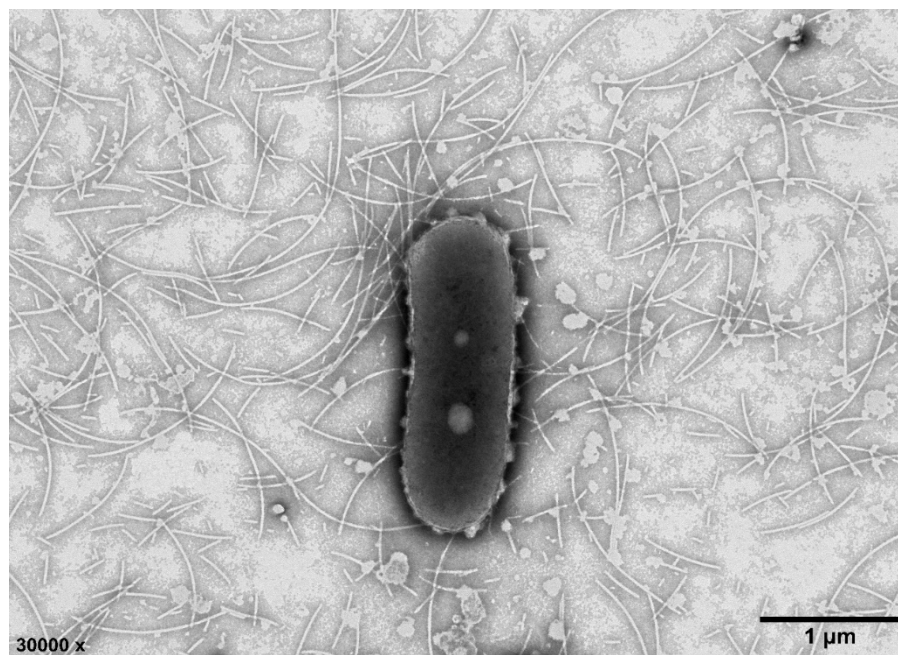

e

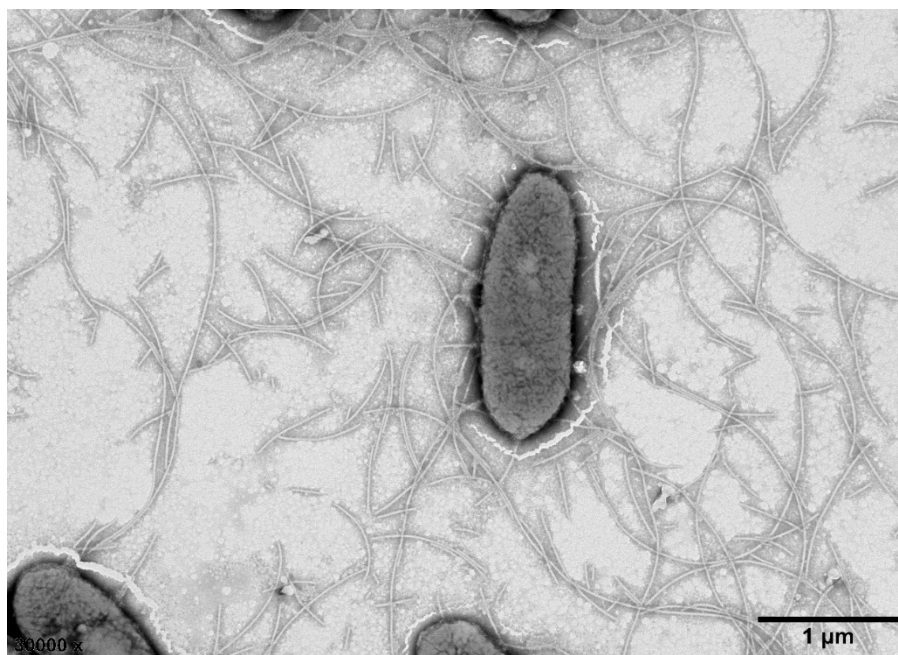

f

Supplement: Supplementary Figure 1 — Genome-based phylogenomic tree of the 14 strains along with 193 other Halomonas genomes and occurrence of genes for nitrogen, sulfur, and other metabolism. Phylogenomic tree was constructed using PhyloPhlAn 3.0. Strains shown in red are organisms isolated in this study. The visualization, annotation, and management of the phylogenomic trees were performed using the web-based tool evolview v3. Type strains of Marinobacter hydrocarbonoclasticus ATCC 49840T, Alcanivorax dieselolei B5T, and Marinospirillum minutulum DSM 6287T were used as outgroup. The 31 right-most columns represent the distribution of key functional genes for nitrogen, sulfur, and other metabolism including carbon metabolism as well as stress responses. Blue represents the denitrification pathway; tan represents regulators for denitrification; green represents assimilation of nitrate and nitrite; fuchsia represents regulators for assimilation of nitrate and nitrite; orange represents the sulfur oxidation pathway; olive represents the PHA synthesis pathway; yellow represents phosphonate transporters and metabolism; dodgerblue represents the ectoine and glycine betaine synthesis pathway; gray represents arsenical oxidation-, reduction-, and resistance-related genes. [file Data_Sheet_1.zip › Figure S1-S16/Figure S3.pdf]

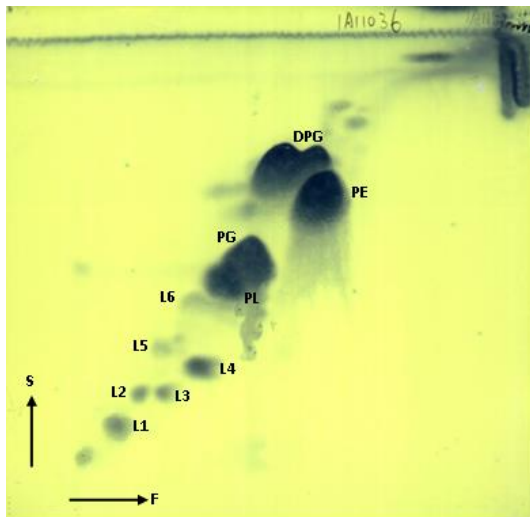

a

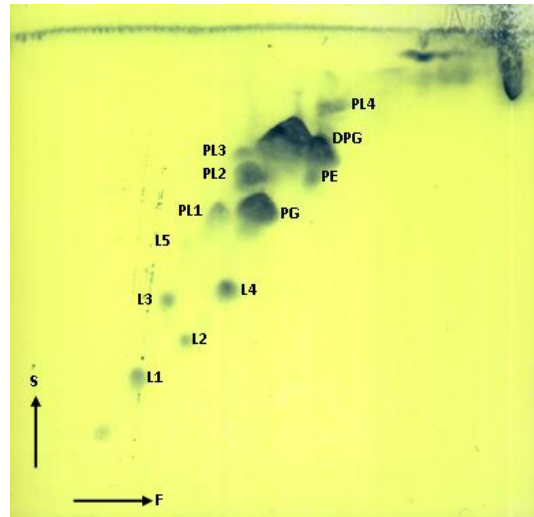

b

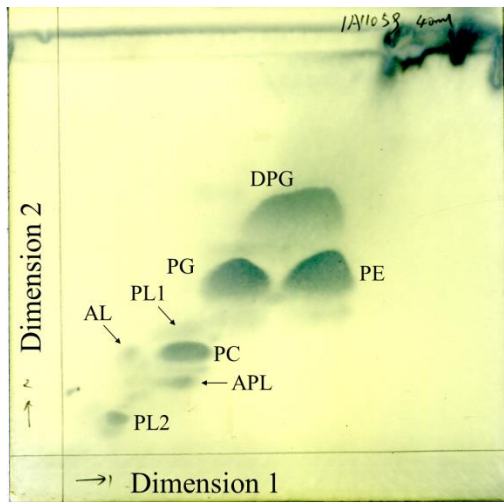

c

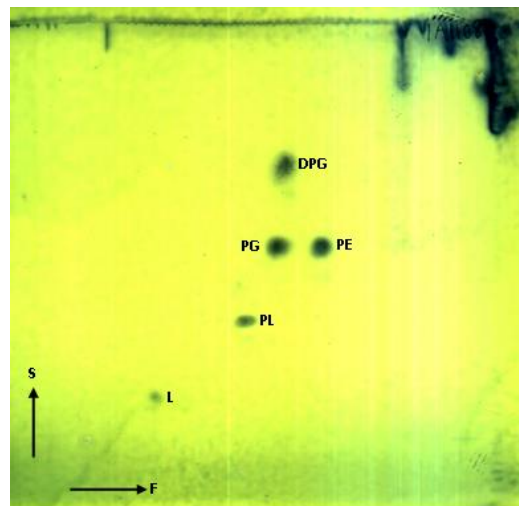

d

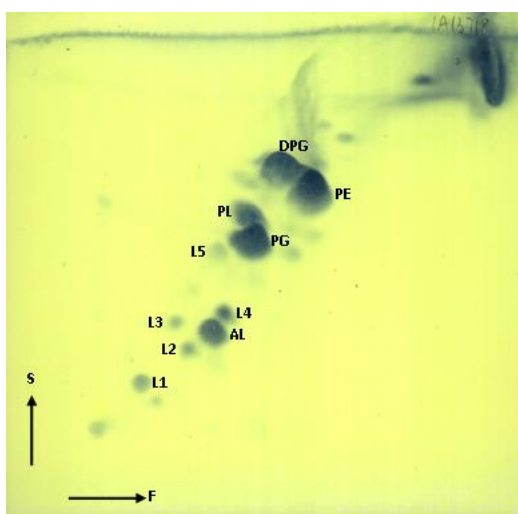

e

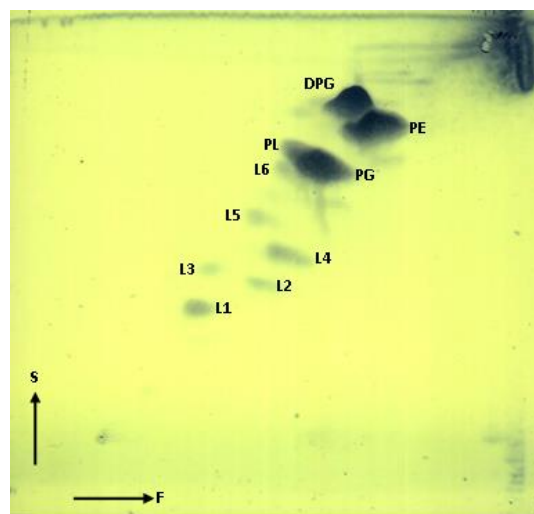

f

Supplement: Supplementary Figure 1 — Genome-based phylogenomic tree of the 14 strains along with 193 other Halomonas genomes and occurrence of genes for nitrogen, sulfur, and other metabolism. Phylogenomic tree was constructed using PhyloPhlAn 3.0. Strains shown in red are organisms isolated in this study. The visualization, annotation, and management of the phylogenomic trees were performed using the web-based tool evolview v3. Type strains of Marinobacter hydrocarbonoclasticus ATCC 49840T, Alcanivorax dieselolei B5T, and Marinospirillum minutulum DSM 6287T were used as outgroup. The 31 right-most columns represent the distribution of key functional genes for nitrogen, sulfur, and other metabolism including carbon metabolism as well as stress responses. Blue represents the denitrification pathway; tan represents regulators for denitrification; green represents assimilation of nitrate and nitrite; fuchsia represents regulators for assimilation of nitrate and nitrite; orange represents the sulfur oxidation pathway; olive represents the PHA synthesis pathway; yellow represents phosphonate transporters and metabolism; dodgerblue represents the ectoine and glycine betaine synthesis pathway; gray represents arsenical oxidation-, reduction-, and resistance-related genes. [file Data_Sheet_1.zip › Figure S1-S16/Figure S4.pdf]

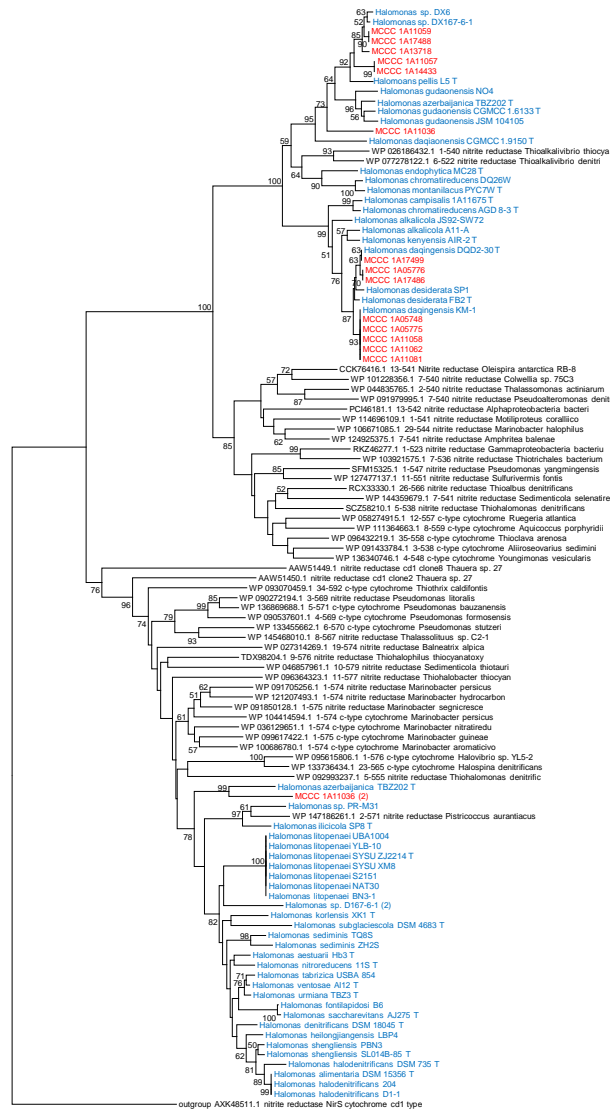

Clade I

Clade II

Supplement: Supplementary Figure 1 — Genome-based phylogenomic tree of the 14 strains along with 193 other Halomonas genomes and occurrence of genes for nitrogen, sulfur, and other metabolism. Phylogenomic tree was constructed using PhyloPhlAn 3.0. Strains shown in red are organisms isolated in this study. The visualization, annotation, and management of the phylogenomic trees were performed using the web-based tool evolview v3. Type strains of Marinobacter hydrocarbonoclasticus ATCC 49840T, Alcanivorax dieselolei B5T, and Marinospirillum minutulum DSM 6287T were used as outgroup. The 31 right-most columns represent the distribution of key functional genes for nitrogen, sulfur, and other metabolism including carbon metabolism as well as stress responses. Blue represents the denitrification pathway; tan represents regulators for denitrification; green represents assimilation of nitrate and nitrite; fuchsia represents regulators for assimilation of nitrate and nitrite; orange represents the sulfur oxidation pathway; olive represents the PHA synthesis pathway; yellow represents phosphonate transporters and metabolism; dodgerblue represents the ectoine and glycine betaine synthesis pathway; gray represents arsenical oxidation-, reduction-, and resistance-related genes. [file Data_Sheet_1.zip › Figure S1-S16/Figure S5.pdf]

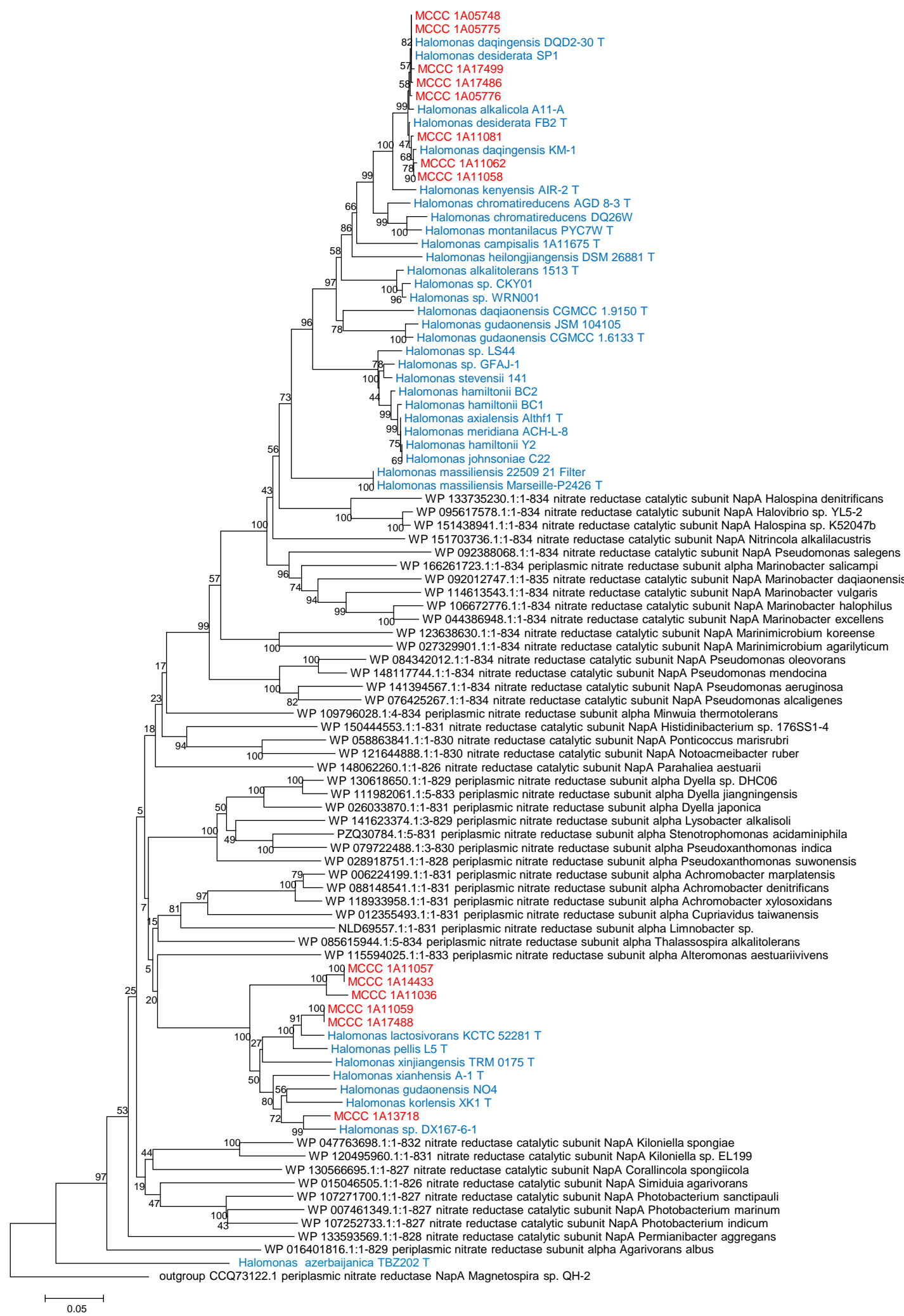

0.05

Supplement: Supplementary Figure 1 — Genome-based phylogenomic tree of the 14 strains along with 193 other Halomonas genomes and occurrence of genes for nitrogen, sulfur, and other metabolism. Phylogenomic tree was constructed using PhyloPhlAn 3.0. Strains shown in red are organisms isolated in this study. The visualization, annotation, and management of the phylogenomic trees were performed using the web-based tool evolview v3. Type strains of Marinobacter hydrocarbonoclasticus ATCC 49840T, Alcanivorax dieselolei B5T, and Marinospirillum minutulum DSM 6287T were used as outgroup. The 31 right-most columns represent the distribution of key functional genes for nitrogen, sulfur, and other metabolism including carbon metabolism as well as stress responses. Blue represents the denitrification pathway; tan represents regulators for denitrification; green represents assimilation of nitrate and nitrite; fuchsia represents regulators for assimilation of nitrate and nitrite; orange represents the sulfur oxidation pathway; olive represents the PHA synthesis pathway; yellow represents phosphonate transporters and metabolism; dodgerblue represents the ectoine and glycine betaine synthesis pathway; gray represents arsenical oxidation-, reduction-, and resistance-related genes. [file Data_Sheet_1.zip › Figure S1-S16/Figure S6.pdf]

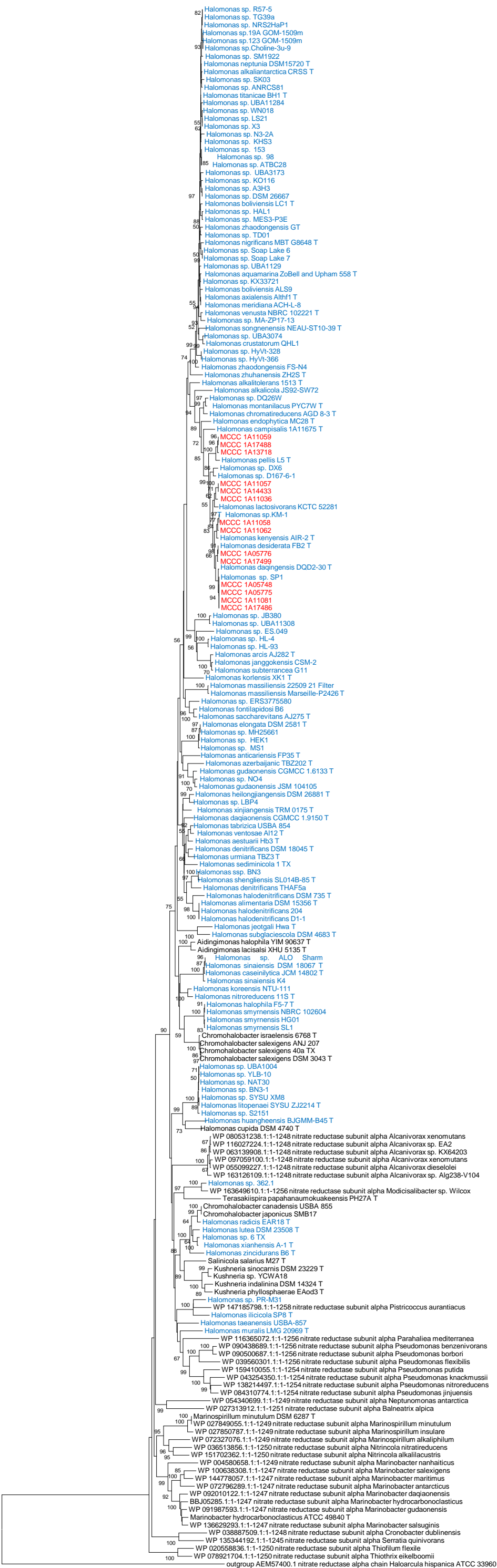

0.1

Supplement: Supplementary Figure 1 — Genome-based phylogenomic tree of the 14 strains along with 193 other Halomonas genomes and occurrence of genes for nitrogen, sulfur, and other metabolism. Phylogenomic tree was constructed using PhyloPhlAn 3.0. Strains shown in red are organisms isolated in this study. The visualization, annotation, and management of the phylogenomic trees were performed using the web-based tool evolview v3. Type strains of Marinobacter hydrocarbonoclasticus ATCC 49840T, Alcanivorax dieselolei B5T, and Marinospirillum minutulum DSM 6287T were used as outgroup. The 31 right-most columns represent the distribution of key functional genes for nitrogen, sulfur, and other metabolism including carbon metabolism as well as stress responses. Blue represents the denitrification pathway; tan represents regulators for denitrification; green represents assimilation of nitrate and nitrite; fuchsia represents regulators for assimilation of nitrate and nitrite; orange represents the sulfur oxidation pathway; olive represents the PHA synthesis pathway; yellow represents phosphonate transporters and metabolism; dodgerblue represents the ectoine and glycine betaine synthesis pathway; gray represents arsenical oxidation-, reduction-, and resistance-related genes. [file Data_Sheet_1.zip › Figure S1-S16/Figure S7.pdf]

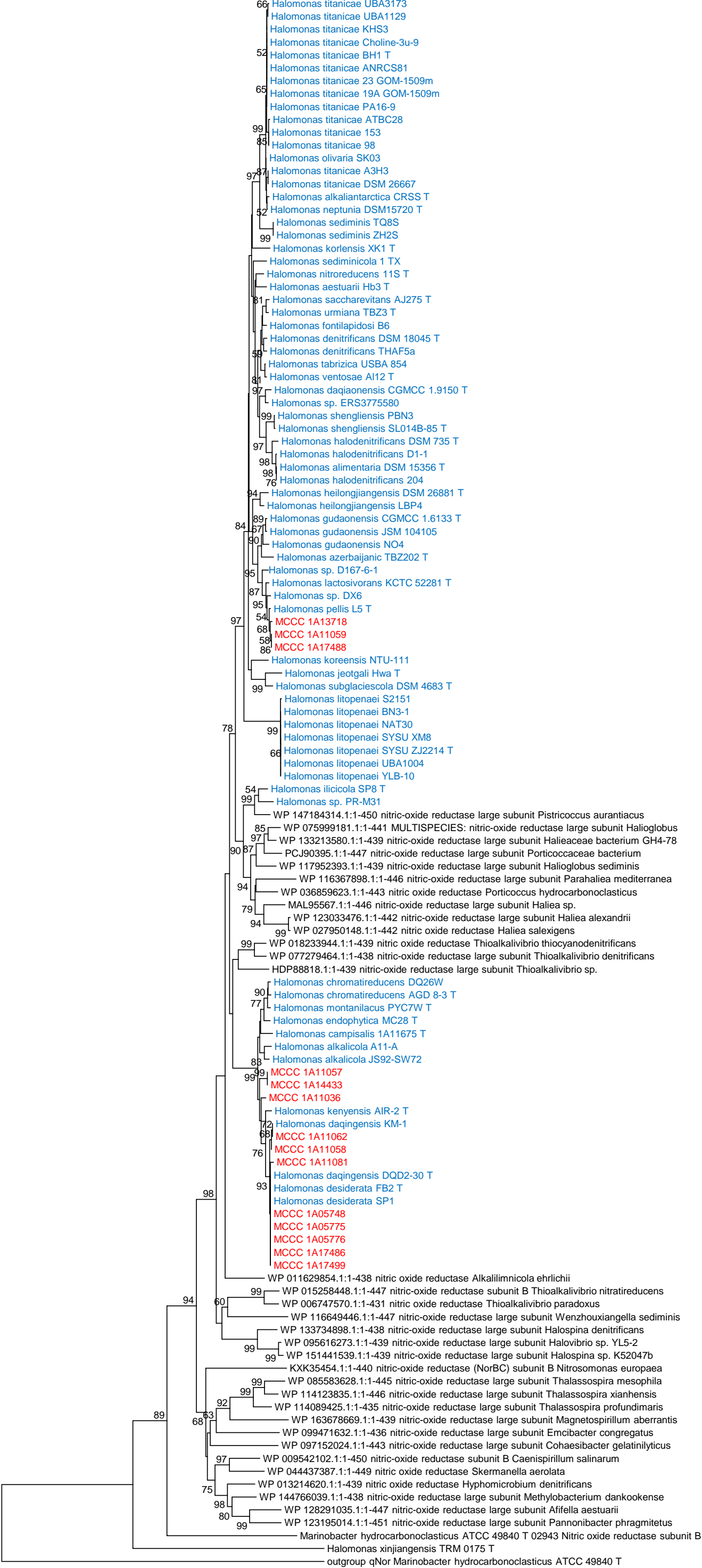

0.1

Supplement: Supplementary Figure 1 — Genome-based phylogenomic tree of the 14 strains along with 193 other Halomonas genomes and occurrence of genes for nitrogen, sulfur, and other metabolism. Phylogenomic tree was constructed using PhyloPhlAn 3.0. Strains shown in red are organisms isolated in this study. The visualization, annotation, and management of the phylogenomic trees were performed using the web-based tool evolview v3. Type strains of Marinobacter hydrocarbonoclasticus ATCC 49840T, Alcanivorax dieselolei B5T, and Marinospirillum minutulum DSM 6287T were used as outgroup. The 31 right-most columns represent the distribution of key functional genes for nitrogen, sulfur, and other metabolism including carbon metabolism as well as stress responses. Blue represents the denitrification pathway; tan represents regulators for denitrification; green represents assimilation of nitrate and nitrite; fuchsia represents regulators for assimilation of nitrate and nitrite; orange represents the sulfur oxidation pathway; olive represents the PHA synthesis pathway; yellow represents phosphonate transporters and metabolism; dodgerblue represents the ectoine and glycine betaine synthesis pathway; gray represents arsenical oxidation-, reduction-, and resistance-related genes. [file Data_Sheet_1.zip › Figure S1-S16/Figure S8.pdf]

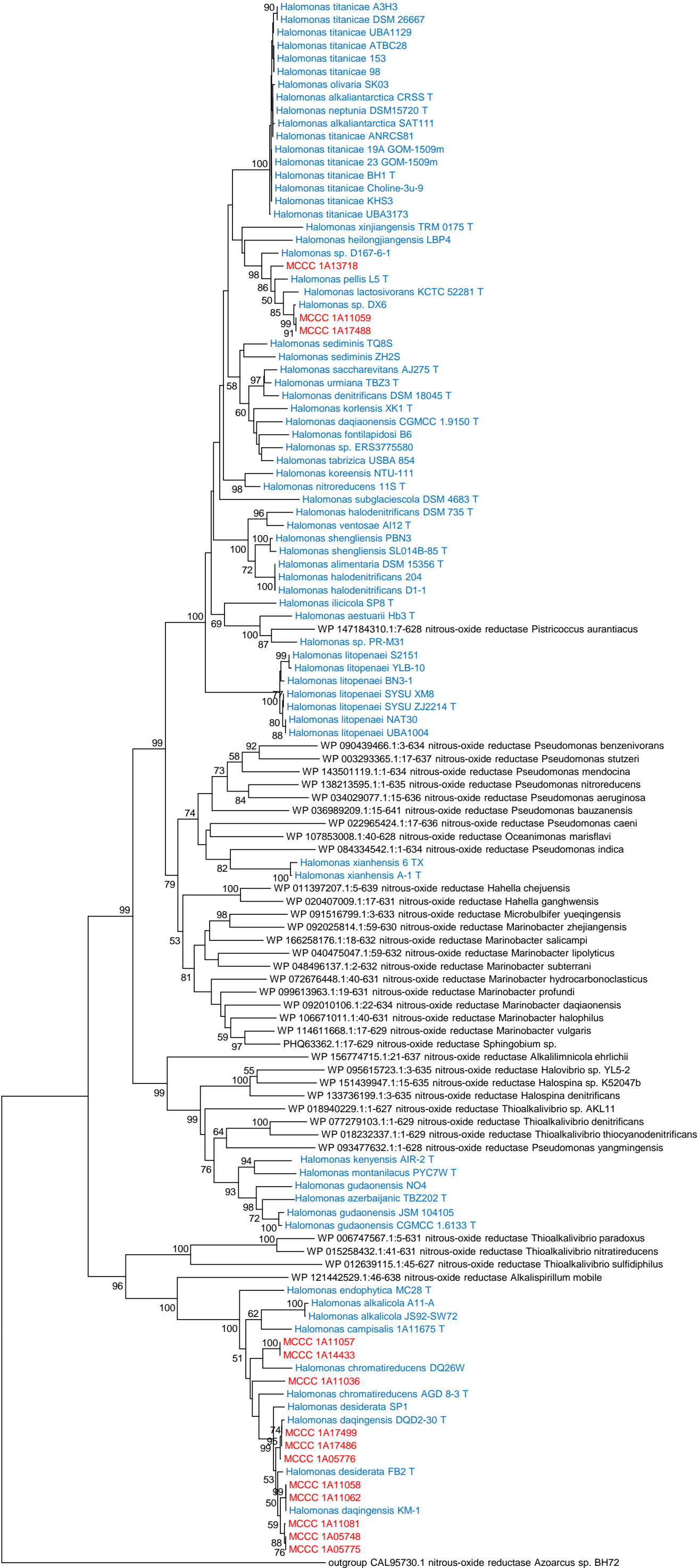

0.05

Supplement: Supplementary Figure 1 — Genome-based phylogenomic tree of the 14 strains along with 193 other Halomonas genomes and occurrence of genes for nitrogen, sulfur, and other metabolism. Phylogenomic tree was constructed using PhyloPhlAn 3.0. Strains shown in red are organisms isolated in this study. The visualization, annotation, and management of the phylogenomic trees were performed using the web-based tool evolview v3. Type strains of Marinobacter hydrocarbonoclasticus ATCC 49840T, Alcanivorax dieselolei B5T, and Marinospirillum minutulum DSM 6287T were used as outgroup. The 31 right-most columns represent the distribution of key functional genes for nitrogen, sulfur, and other metabolism including carbon metabolism as well as stress responses. Blue represents the denitrification pathway; tan represents regulators for denitrification; green represents assimilation of nitrate and nitrite; fuchsia represents regulators for assimilation of nitrate and nitrite; orange represents the sulfur oxidation pathway; olive represents the PHA synthesis pathway; yellow represents phosphonate transporters and metabolism; dodgerblue represents the ectoine and glycine betaine synthesis pathway; gray represents arsenical oxidation-, reduction-, and resistance-related genes. [file Data_Sheet_1.zip › Figure S1-S16/Figure S9.pdf]
