# Supplementary material for: Aerobic Denitrification and Heterotrophic Sulfur Oxidation in the Genus Halomonas Revealed by Six Novel Species Characterizations and Genome-Based Analysis
Source: Front Microbiol. 2021 Mar 18;12:652766. doi: 10.3389/fmicb.2021.652766 (PMC8014003; doi:10.3389/fmicb.2021.652766)
Supplement: Supplementary Figure 1 — Genome-based phylogenomic tree of the 14 strains along with 193 other Halomonas genomes and occurrence of genes for nitrogen, sulfur, and other metabolism. Phylogenomic tree was constructed using PhyloPhlAn 3.0. Strains shown in red are organisms isolated in this study. The visualization, annotation, and management of the phylogenomic trees were performed using the web-based tool evolview v3. Type strains of Marinobacter hydrocarbonoclasticus ATCC 49840T, Alcanivorax dieselolei B5T, and Marinospirillum minutulum DSM 6287T were used as outgroup. The 31 right-most columns represent the distribution of key functional genes for nitrogen, sulfur, and other metabolism including carbon metabolism as well as stress responses. Blue represents the denitrification pathway; tan represents regulators for denitrification; green represents assimilation of nitrate and nitrite; fuchsia represents regulators for assimilation of nitrate and nitrite; orange represents the sulfur oxidation pathway; olive represents the PHA synthesis pathway; yellow represents phosphonate transporters and metabolism; dodgerblue represents the ectoine and glycine betaine synthesis pathway; gray represents arsenical oxidation-, reduction-, and resistance-related genes. [file Figure S1.pdf › Figure S1-S16/Figure S1.pdf]

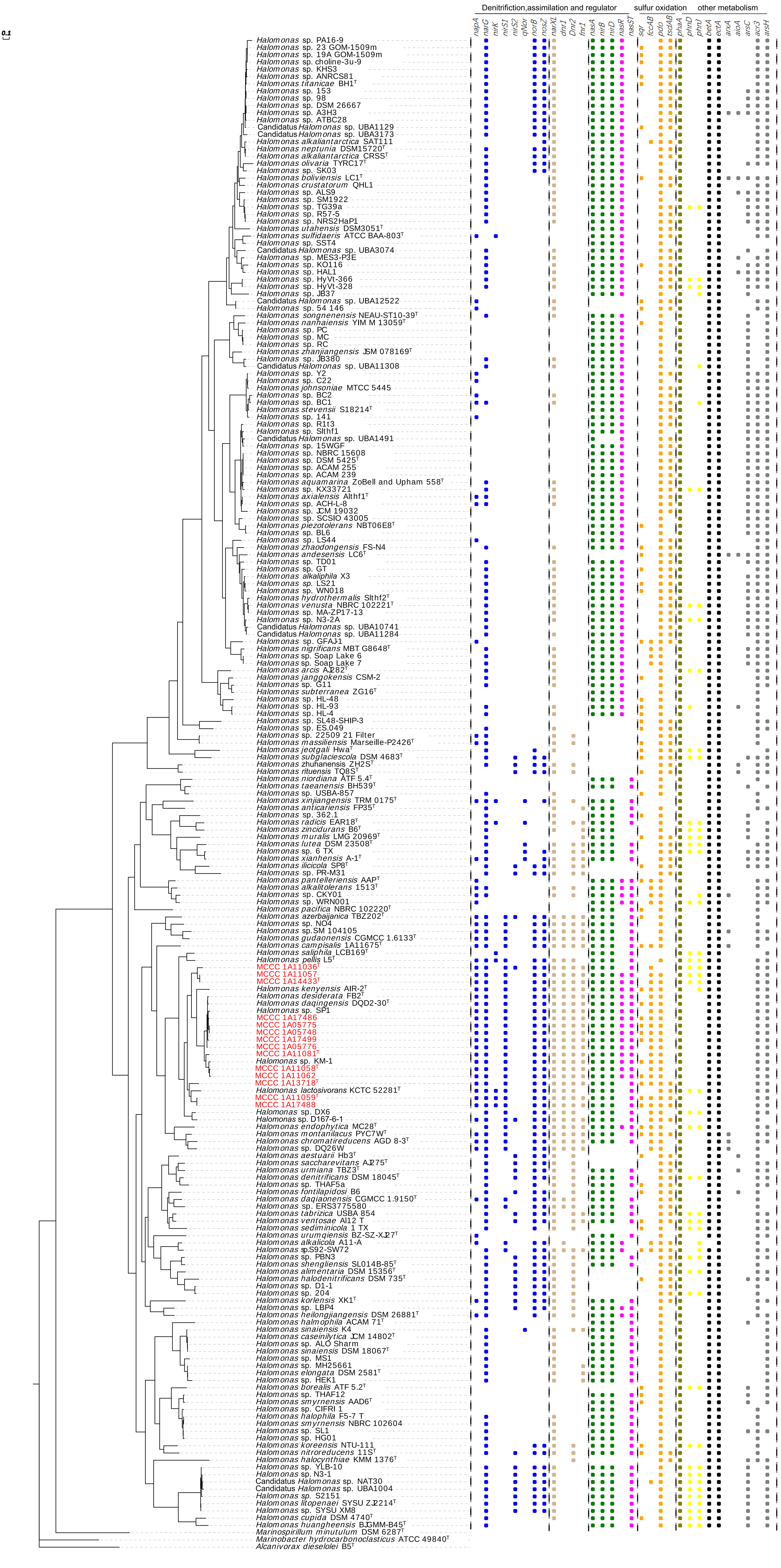
0.1
Denitrifiction,assimilation and regulator
sulfur oxidation
other metabolism
napA
narG
nirK
nirS1
nirS2
qNor
norB
nosZ
narXL
dhr1
Dnr2
fnr1
nasA
nirB
nirD
nasR
nasST
sqr
fccAB
pdo
tsdAB
phaA
phnD
phnJ
betA
ectA
arxA
aioA
arsC
acr3
arsH
Halomonas sp. PA16-9
Halomonas sp. 23 GOM-1509m
Halomonas sp. 19A GOM-1509m
Halomonas sp. choline-3u-9
Halomonas sp. KHS3
Halomonas sp. ANRCS81
Halomonas titanicae BH1^{T}
Halomonas sp. 153
Halomonas sp. 98
Halomonas sp. DSM 26667
Halomonas sp. A3H3
Halomonas sp. ATBC28
Candidatus Halomonas sp. UBA1129
Candidatus Halomonas sp. UBA3173
Halomonas alkaliantarctica SAT111
Halomonas neptunia DSM15720^{T}
Halomonas alkaliantarctica CRSS^{T}
Halomonas olivaria TYRC17^{T}
Halomonas sp. SK03
Halomonas boliviensis LC1^{T}
Halomonas crustatorum QHL1
Halomonas sp. ALS9
Halomonas sp. SM1922
Halomonas sp. TG39a
Halomonas sp. R57-5
Halomonas sp. NRS2HaP1
Halomonas utahensis DSM3051^{T}
Halomonas sulfidaeris ATCC BAA-803^{T}
Halomonas sp. SST4
Candidatus Halomonas sp. UBA3074
Halomonas sp. MES3-P3E
Halomonas sp. KO116
Halomonas sp. HAL1
Halomonas sp. HyVt-366
Halomonas sp. HyVt-328
Halomonas sp. JB37
Candidatus Halomonas sp. UBA12522
Halomonas sp. 54 146
Halomonas songnenensis NEAU-ST10-39^{T}
Halomonas nanhaiensis YIM M 13059^{T}
Halomonas sp. PC
Halomonas sp. MC
Halomonas sp. RC
Halomonas zhanjiangensis JSM 078169^{T}
Halomonas sp. JB380
Candidatus Halomonas sp. UBA11308
Halomonas sp. Y2
Halomonas sp. C22
Halomonas johnsoniae MTCC 5445
Halomonas sp. BC2
Halomonas sp. BC1
Halomonas stevensii S18214^{T}
Halomonas sp. 141
Halomonas sp. R1t3
Halomonas sp. Slthf1
Candidatus Halomonas sp. UBA1491
Halomonas sp. 15WGF
Halomonas sp. NBRC 15608
Halomonas sp. DSM 5425^{T}
Halomonas sp. ACAM 255
Halomonas sp. ACAM 239
Halomonas aquamarina ZoBell and Upham 558^{T}
Halomonas sp. KX33721
Halomonas axialensis Althf1^{T}
Halomonas sp. ACH-L-8
Halomonas sp. JCM 19032
Halomonas sp. SCSIO 43005
Halomonas piezotolerans NBT06E8^{T}
Halomonas sp. BL6
Halomonas sp. LS44
Halomonas zhaodongensis FS-N4
Halomonas andesensis LC6^{T}
Halomonas sp. TD01
Halomonas sp. GT
Halomonas alkaliphila X3
Halomonas sp. LS21
Halomonas sp. WN018
Halomonas hydrothermalis Slthf2^{T}
Halomonas venusta NBRC 102221^{T}
Halomonas sp. MA-ZP17-13
Halomonas sp. N3-2A
Candidatus Halomonas sp. UBA10741
Candidatus Halomonas sp. UBA11284
Halomonas sp. GFAJ-1
Halomonas nigrificans MBT G8648^{T}
Halomonas sp. Soap Lake 6
Halomonas sp. Soap Lake 7
Halomonas arcis AJ282^{T}
Halomonas janggokensis CSM-2
Halomonas sp. G11
Halomonas subterranea ZG16^{T}
Halomonas sp. HL-48
Halomonas sp. HL-93
Halomonas sp. HL-4
Halomonas sp. SL48-SHIP-3
Halomonas sp. ES.049
Halomonas sp. 22509 21 Filter
Halomonas massiliensis Marseille-P2426^{T}
Halomonas jeotgali Hwa^{T}
Halomonas subglaciescola DSM 4683^{T}
Halomonas zhuhanensis ZH2S^{T}
Halomonas rituensis TQ8S^{T}
Halomonas niordiana ATF 5.4^{T}
Halomonas taeanensis BH539^{T}
Halomonas sp. USBA-857
Halomonas xinjiangensis TRM 0175^{T}
Halomonas anticariensis FP35^{T}
Halomonas sp. 362.1
Halomonas radicis EAR18^{T}
Halomonas zincidurans B6^{T}
Halomonas muralis LMG 20969^{T}
Halomonas lutea DSM 23508^{T}
Halomonas sp. 6 TX
Halomonas xianhensis A-1^{T}
Halomonas ilicicola SP8^{T}
Halomonas sp. PR-M31
Halomonas pantelleriensis AAP^{T}
Halomonas alkalitolerans 1513^{T}
Halomonas sp. CKY01
Halomonas sp. WRN001
Halomonas pacifica NBRC 102220^{T}
Halomonas azerbaijanica TBZ202^{T}
Halomonas sp. NO4
Halomonas sp.SM 104105
Halomonas gudaonensis CGMCC 1.6133^{T}
Halomonas campisalis 1A11675^{T}
Halomonas saliphila LCB169^{T}
Halomonas pellis L5^{T}
MCCC 1A11036^{T}
MCCC 1A11057
MCCC 1A14433^{T}
Halomonas kenyensis AIR-2^{T}
Halomonas desiderata FB2^{T}
Halomonas daqingensis DQD2-30^{T}
Halomonas sp. SP1
MCCC 1A17486
MCCC 1A05775
MCCC 1A05748
MCCC 1A17499
MCCC 1A05776
MCCC 1A11081^{T}
Halomonas sp. KM-1
MCCC 1A11058^{T}
MCCC 1A11062
MCCC 1A13718^{T}
Halomonas lactosivorans KCTC 52281^{T}
MCCC 1A11059^{T}
MCCC 1A17488
Halomonas sp. DX6
Halomonas sp. D167-6-1
Halomonas endophytica MC28^{T}
Halomonas montanilacus PYC7W^{T}
Halomonas chromatireducens AGD 8-3^{T}
Halomonas sp. DQ26W
Halomonas aestuarii Hb3^{T}
Halomonas saccharevitans AJ275^{T}
Halomonas urmiana TBZ3^{T}
Halomonas denitrificans DSM 18045^{T}
Halomonas sp. THAF5a
Halomonas fontilapidosi B6
Halomonas daqiaonensis CGMCC 1.9150^{T}
Halomonas sp. ERS3775580
Halomonas tabrizica USBA 854
Halomonas ventosae Al12 T
Halomonas sediminicola 1 TX
Halomonas urumqiensis BZ-SZ-XJ27^{T}
Halomonas alkalicola A11-A
Halomonas sp.S92-SW72
Halomonas sp. PBN3
Halomonas shengliensis SL014B-85^{T}
Halomonas alimentaria DSM 15356^{T}
Halomonas halodenitrificans DSM 735^{T}
Halomonas sp. D1-1
Halomonas sp. 204
Halomonas korlensis XK1^{T}
Halomonas sp. LBP4
Halomonas heilongjiangensis DSM 26881^{T}
Halomonas halmophila ACAM 71^{T}
Halomonas sinaiensis K4
Halomonas caseinilytica JCM 14802^{T}
Halomonas sp. ALO Sharm
Halomonas sinaiensis DSM 18067^{T}
Halomonas sp. MS1
Halomonas sp. MH25661
Halomonas elongata DSM 2581^{T}
Halomonas sp. HEK1
Halomonas borealis ATF 5.2^{T}
Halomonas sp. THAF12
Halomonas smyrnensis AAD6^{T}
Halomonas sp. CIFRI 1
Halomonas halophila F5-7 T
Halomonas smyrnensis NBRC 102604
Halomonas sp. SL1
Halomonas sp. HG01
Halomonas koreensis NTU-111
Halomonas nitroreducens 11S^{T}
Halomonas halocynthiae KMM 1376^{T}
Halomonas sp. YLB-10
Halomonas sp. N3-1
Candidatus Halomonas sp. NAT30
Candidatus Halomonas sp. UBA1004
Halomonas sp. S2151
Halomonas litopenaei SYSU ZJ2214^{T}
Halomonas sp. SYSU XM8
Halomonas cupida DSM 4740^{T}
Halomonas huangheensis BJGMM-B45^{T}
Marinospirillum minutulum DSM 6287^{T}
Marinobacter hydrocarbonoclasticus ATCC 49840^{T}
Alcanivorax dieselolei B5^{T}
